# Genus *Periploca* (Apocynaceae): A Review of Its Classification, Phytochemistry, Biological Activities and Toxicology

**DOI:** 10.3390/molecules24152749

**Published:** 2019-07-29

**Authors:** Mingjin Huang, Shoumao Shen, Chunli Luo, Yan Ren

**Affiliations:** 1 College of Agriculture, Guizhou University, Guiyang 550025, Guizhou, China; 2 State Key Laboratory of Propagation and Cultivation on Medicinal Plants of Guizhou Province, Guiyang 550025, Guizhou, China; 3 School of Pharmacy, Yancheng Teachers’ University, Yancheng 224002, Jiangsu, China; 4 College of Pharmacy, Guizhou University, Guiyang 550025, Guizhou, China

**Keywords:** genus *Periploca*, phytochemistry, biological activities, classification, toxicology, review

## Abstract

The genus *Periploca* belongs to the family Apocynaceae, which is composed of approximately ten species of plants according to incomplete statistics. Most of these plants serve as folk medicines with a long history, especially *Periploca sepium* and *Periploca forrestii*. The botanical classifications, chemical constituents, biological activities and toxicities of the genus *Periploca* were summarized in the literature from 1897 to early 2019. Though the botanical classification of this genus is controversial, these species are well-known to be rich sources of diverse and complex natural products—above all, cardiac steroids and C_21_ pregnane steroids with special structures and obvious pharmacological activities. The various crude extracts and 314 isolated metabolites from this genus have attracted much attention in intensive biological studies, indicating that they are equipped with cardiotonic, anti-inflammatory, immunosuppressive, antitumor, antimicrobial, antioxidant, insecticidal and other properties. It is noteworthy that some cardiac glycosides showed hepatotoxicity and cardiotoxicity at certain doses. Therefore, in view of the medical and agricultural value of the genus *Periploca*, in-depth investigations of the pharmacology in vivo, the mechanisms of biological actions, and the pharmacokinetics of the active ingredients should be carried out in the future. Moreover, in order to ensure the safety of clinical medication, the potential toxicities of cardiac glycosides or other compounds should also be paid attention. This systematic review provides an important reference base for applied research on pharmaceuticals and pesticides from this genus.

## 1. Introduction

**Figure 1:**
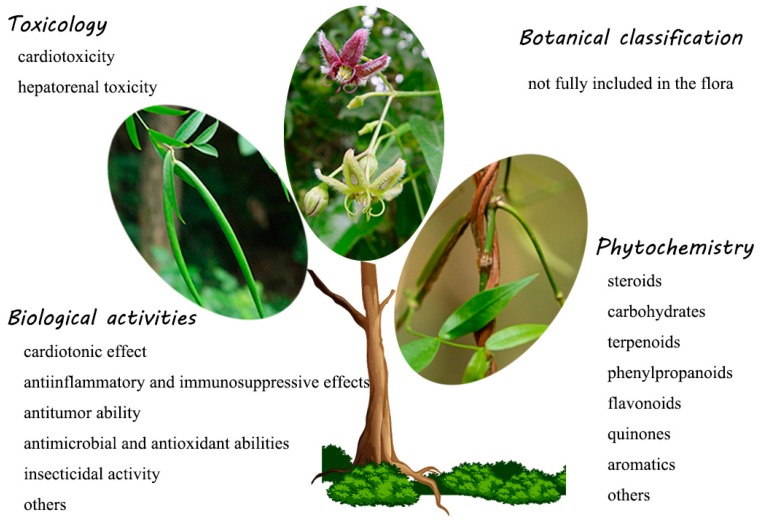
The outline and some representative plants of the genus *Periploca*. (Left: The fruits of *P. forrestii*; Centre: The flowers of *P. sepium*; Right: The stems of *P. forrestii*. The center and right pictures were downloaded from Plant Photo Bank of China, PPBC.).

The genus *Periploca* belongs to the family Apocynaceae [1] and involves approximately ten species which are diffusely distributed in temperate Asia, southern Europe and tropical Africa [2]. Many species of this genus have been historically used as folk medicines in China, especially *Periploca sepium* and *Periploca forrestii*. The root bark of *P. sepium*, known as “*Xiangjiapi*” or “Bei-wujiapi,” is utilized as a traditional Chines medicine (TCM) for the treatment of rheumatoid arthritis and bone and muscle pain, which is recorded in the *Chinese Pharmacopoeia* (2015 version). The processing of Cortex Periplocae requires removing impurities, cleaning, slicing, and drying. The dried Cortex Periplocae (3~6 g) and other medicinal materials are decocted in water and taken for internal use [3]. The stems or the whole plant of *P. forrestii*, which is a well-known herb known as *“*Heiguteng,*”* are widely used by the Miao nationality in China to treat many diseases, including rheumatic arthritis, traumatic injury, stomachache, dyspepsia, and amenorrhea. The usage of *P. forrestii* is usually decocted in water or soaked in wine for internal use, or it is smashed for external application [4]. The title genus is abundant with an array of attractive metabolites, such as steroids, oligosaccharides, terpenoids, phenylpropanoids, and flavonoids. These novel and complex metabolites and various crude extracts from the genus *Periploca* have attracted increasing attention to intensive biological studies, indicating that they possess significant cardiotonic, anti-inflammatory, immunosuppressive, antitumor, antimicrobial, antioxidant, insecticidal and other properties. Some cardenolide-type steroids exhibit obvious cardiotonic and antitumor effects at a certain dose. Their strong immunosuppression and insecticidal potentials provide new development prospects for pregnane glycosides. In addition, cardiac steroids were also found to have latent toxicity. In the 2000s, several researchers provided reviews of the chemical constituents and biological activities of the genus *Periploca* [5,6]. However, these concise reviews were expressed in Chinese. Additionally, a large amount of scientific research results on this genus has emerged in recent years. To make researchers know elaborate information on the chemical and biological activities of the genus *Periploca* and to make a modest contribution to the rational application of this genus, a systematic review of the genus *Periploca* is imperative. We covered topics including the botanical classification, phytochemistry, biological activities and toxicology of the genus *Periploca* through a literature survey from 1897 to early 2019. The outline of this paper and some representative plants of the genus *Periploca* are shown in Figure 1.

## 2. Botanical Classification of the Genus *Periploca*

In 1895, Schumann was the first to classify the *P.* species. Browicz defined the genus *Periploca* and recognized eleven species in 1966. According to *Flora of China* (1995) [2], the genus *Periploca* comprises about ten species, and five species are distributed in China. Additionally, in 1997, Venter [7] suggested correcting the nomenclature of *Periploca* to provide diagnostic characteristics for the genus and to improve classification. However, fourteen species belonging to genus *Periploca* were recorded by Venter, not including *P. forrestii*, which largely grows in southern China. Moreover, several Chinese scholars are interested in the taxonomy of this genus. *Periploca omeiensis* Z. Y. Zhu, which is merely distributed in Emei Mountain, China, was identified as a new species by Zhu in 1991 [8]. Furthermore, another new species, named *Periploca chrysantha* D. S. Yao, X. C. Chen et J. W. Ren, was found in Gansu, China in 2002 and is similar to *P. sepium*, with a difference in the corolla [9]. However, these two new species have not been included in the flora at home and abroad to date. To our best knowledge, the botanical classification of *Periploca* is inconsistent at home and abroad, and the flora has not been updated in time. We did not take into account the disputes over the taxonomy of the genus *Periploca* and summarized all the mentioned plants of this genus which have been studied for phytochemistry and biological activities. The names and references of these thirteen studied species are shown in Table 1.

## 3. Chemical Constituents from the Genus *Periploca*

Phytochemical investigations of *Periploca* species revealed predominant metabolites, such as steroids, carbohydrates, terpenoids, phenylpropanoids, flavonoids, quinones, and aromatics. To date, three hundred and fourteen compounds (**1**–**314**) have been isolated and identified from the plants of genus *Periploca.*

### 3.1. Steroids

**Figure 2:**
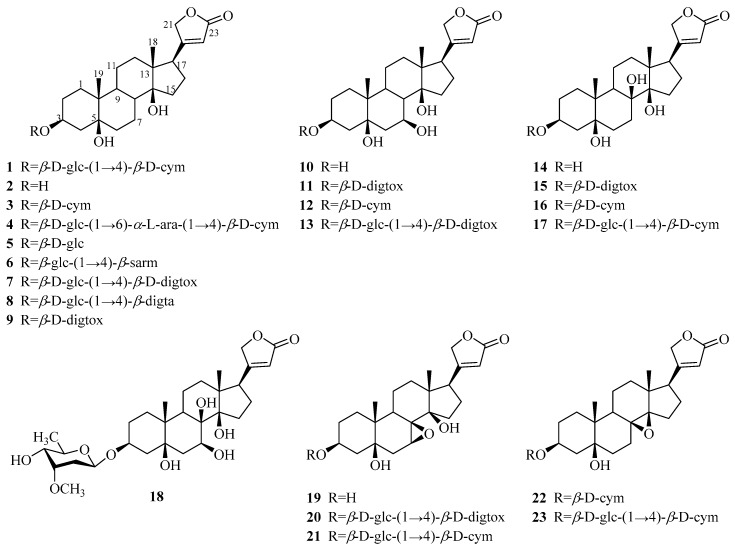

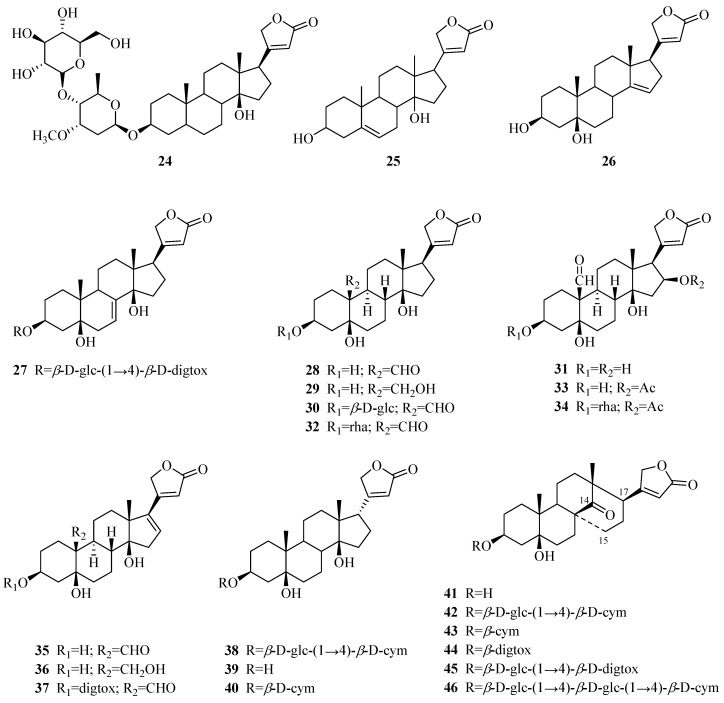
Cardenolide-type steroids from the genus *Periploca*.

**Figure 3:**
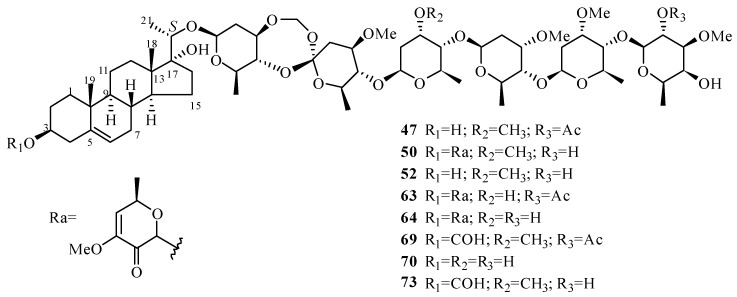

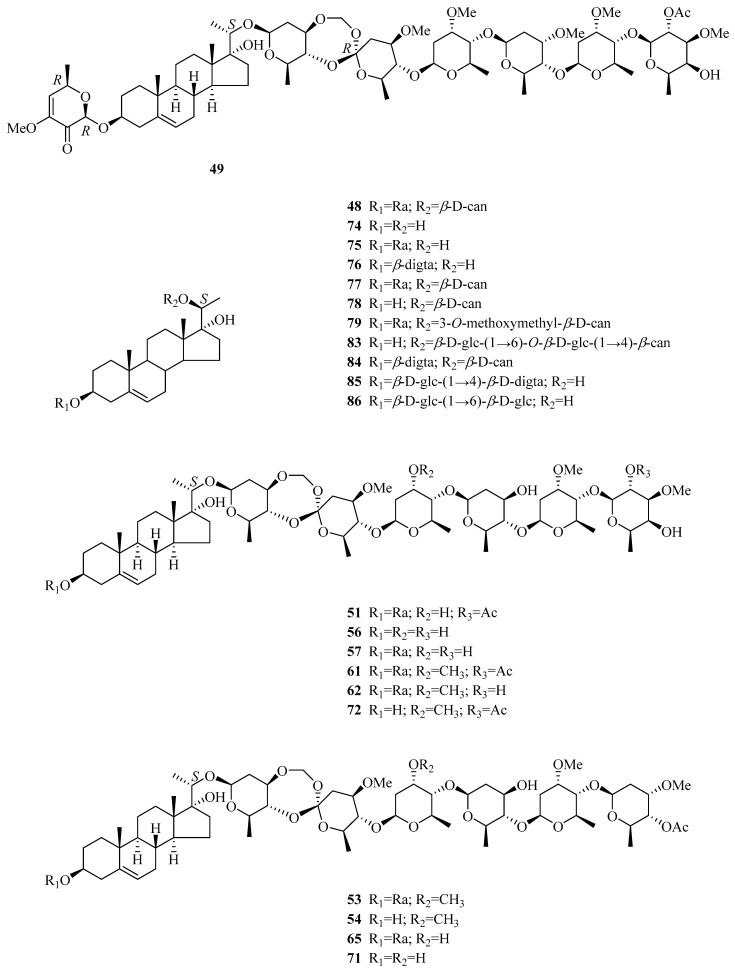

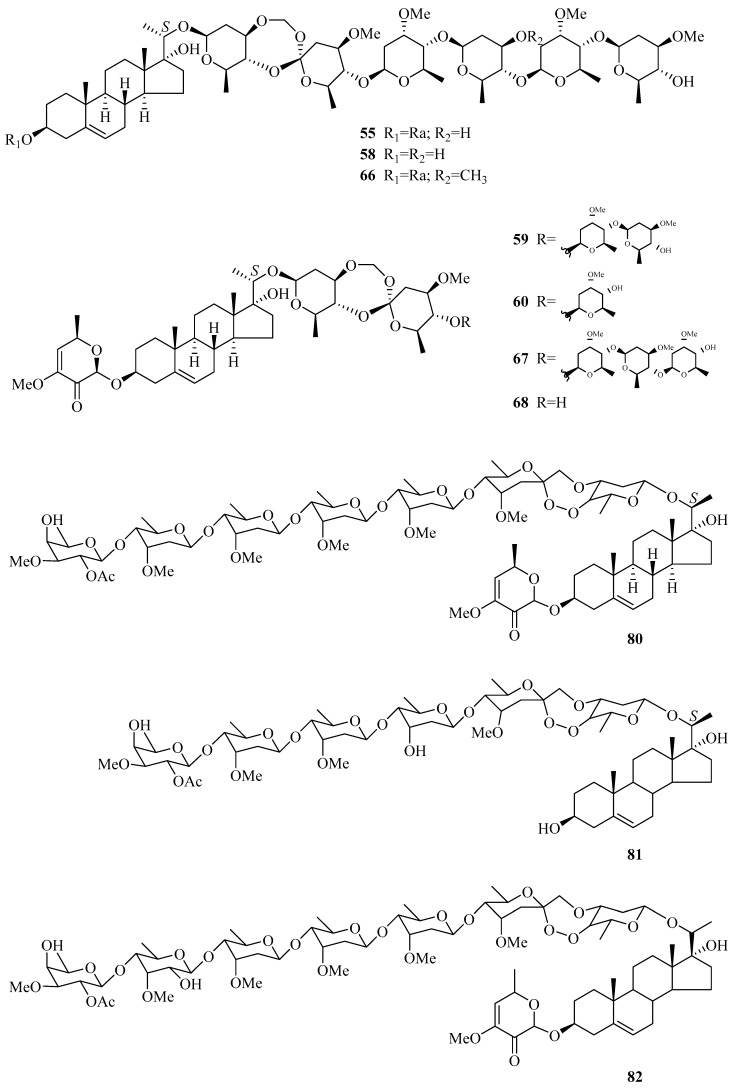

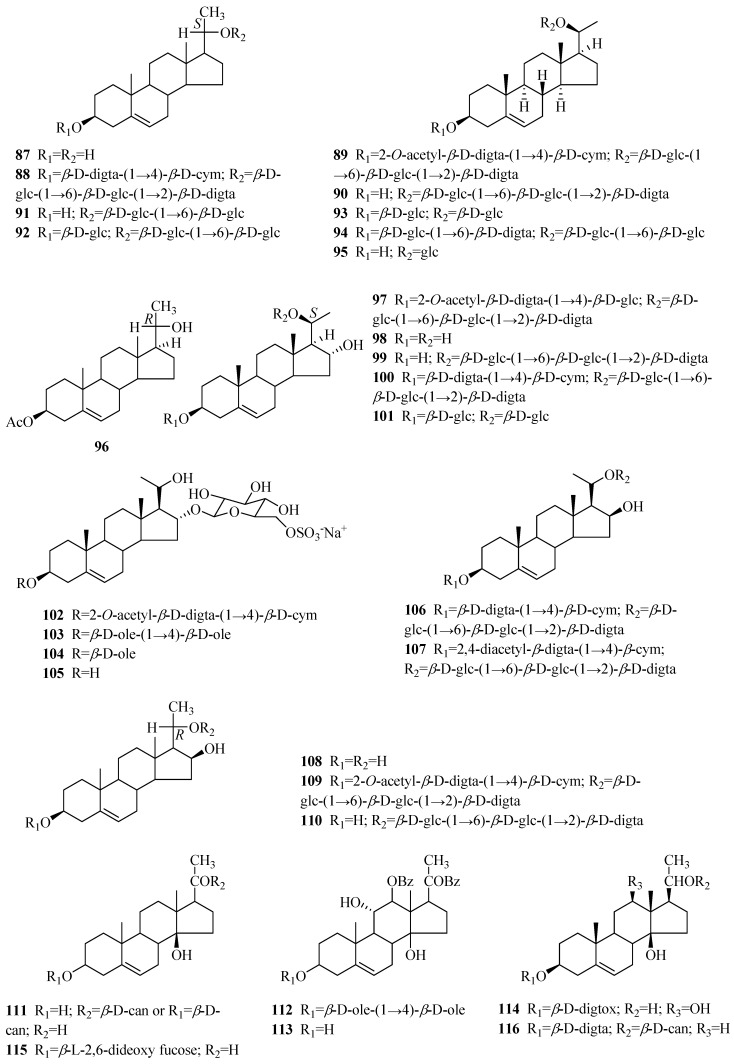

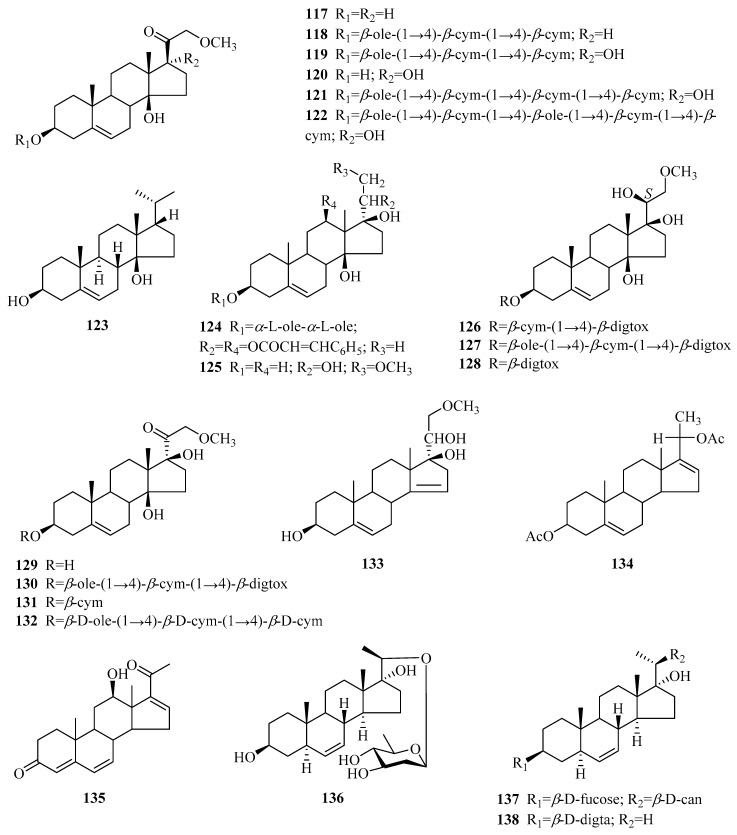
C_21_ pregnane-type steroids from the genus *Periploca*.

**Figure 4:**
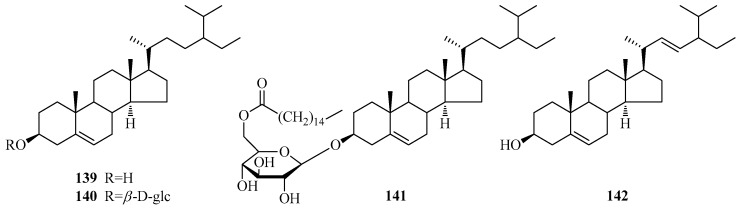
Phytosterol-type steroids from the genus *Periploca*.

One hundred and forty-two steroids (**1**–**142**) were isolated from *Periploca* species, including forty-six cardenolides (**1**–**46**), ninety-two C_21_ pregnane-type steroids (**47**–**138**), and four phytosterols (**139**–**142**). Among them, approximately 80% of the C_21_ pregnane-type steroids come from *P. sepium*, while *P. forrestii* is a rich source of cardenolides. Their structures, names, corresponding sources, parts of plants and references are illuminated in Table 2 and Figure 2, Figure 3 and Figure 4.

#### 3.1.1. Cardenolides

The chemical investigation of this genus led to the discovery of cardenolides. According to their structural skeletons, forty-six cardenolides (**1**–**46**) are classified into two categories, namely periplogenin-type cardenolides (**1**–**40**) and periforgenin A-type cardenolides (**41**–**46**). Among them, there are fourteen aglycones, sixteen monosaccharide glycosides, fourteen disaccharide glycosides, and two trisaccharide glycosides. It is worth noting that all glycosides possess a sugar chain only at the C-3 position. Additionally, every disaccharide glycoside contains a deoxysugar as the inner sugar and a moiety of glucose as the outer sugar, with a linkage mode of 1→4, while the sugar unit of monosaccharide glycosides is a deoxygenated or an oxygenated sugar.

The isolation and identification of periplocin (**1**), the first cardenolide with digitoxin-like efficacy, was reported by Lehmann in 1897 [11]. Periplocin (**1**) was contained in the bark of *P. graeca* found in the southwest Caucasus. Upon heating the glucoside periplocin with dilute sulphuric acid, periplogenin (**2**) [11] was obtained. In 1939, Stoll and co-workers discovered that the enzymatic hydrolysis of periplocin with strophanthobiase produced periplocymarin (**3**) [17]. Later, compounds **1**–**3** were also repeatedly isolated from other species of genus *Periploca* [8,12,13,14,16]. Compared with periplocin, compounds **4**–**9** [15,18,19,20] have the same structural characteristics in their aglycones, with a difference in the sugar chains at C-3. Yu and co-workers [19,20] identified a series of new cardiac glycosides by online analysis or analysis after separation from the stems of *P. forrestii*. During this work, compounds **10**–**17** were found to have a hydroxyl group in the *β* configuration at C-7 or C-8, while a new compound (**18**) was found with two hydroxyl groups in the *β* configuration at C-7 and C-8. In addition, three new 7,8-*β*-epoxy cardiac glycosides (**19**–**21**) were designated. It is worth mentioning that a substitution at C-7 and C-8 led to a decrease of cytotoxic activity. Two new cardiac glycosides with 8,14-*β*-epoxy groups were isolated and identified to be periploforgeside A and periploforgeside B (**22**,**23**) [22]. Echubioside (**24**) [15], without the 5-*β*-hydroxyl group, has a logPo/w value (3.6) similar to that of estradiol (3.3), as determined in the same set of experiments. Compounds **25**–**27** are characterized by a double bond at position Δ^5,6^, Δ^14,15^ or Δ^7,8^ [19]. In 1954, the chemical investigation of *P. nigrescens* resulted in strophanthidin (**28**) and strophanthidol (**29**), with both containing an oxygenic substituent at C-19 [24]. Later, in 1957 and 1965, eight analogues (**30**–**37**) were isolated from the same species [25,26]. Compounds **35**–**37** are reduced by a pair of hydrogen atoms to form a double bond at C-16 and C-17 compared with **28** and **29**. Compounds **38**–**40** are characteristic of 17α-cardenolides [15].

The study of pharmacologically active ingredients from *P. forrestii* led to the discovery of periforgenin A and periforoside I (**41**,**42**), displaying a 15(14→8)abeo-(8*S*)-14-ketone-card skeleton characterized by a ketone at C-14 and a transformed C/D ring [28]. The structure of periforoside I was supported by X-ray diffraction in 1990. Four newly found cardiac glycosides (**43**–**46**) [19,20,29,30] have different structural characteristics in the saccharide chains. The six distinct periforgenin A-type cardiac glycosides were only found in *P. forrestii*, which is the difference between *P. forrestii* and other plants of the genus.

#### 3.1.2. C_21_ Pregnane-Type Steroids

According to the structural characteristics of the ninety-two C_21_ pregnane-type steroids (**47**–**138**, Table 2, Figure 3), a simple conclusion can be summarized. Up to now, all C_21_ steroids from *Periploca* species have possessed a typical pregnane unit with four unbroken rings. Most exist in the form of glycosides, with the sugar chains linked to C-3 or C-20. These saccharide chains are composed of oxygenated sugars, deoxygenated sugars, and their derivatives. Some oxygen-containing groups, such as hydroxy and methoxy groups, are mostly present at the C-3, C-14, C-16, C-17, C-20 and C-21 positions of the aglycone. Additionally, the absolute configuration at C-20 of most compounds (**47**–**81**,**87**–**92**, **97**–**101**,**126**–**128**) has been deduced to be *S*.

From 1967 to 1995, some Japanese researchers made great contributions to the chemical investigation of *P. sepium*. They isolated and identified many compounds with novel features, especially a series of pregnane glycosides. In 1987 and 1988, Itokawa and co-workers [31,36,37,41] discovered periplocosides A–K, all containing a peroxy function, from the antitumor fraction of the CHCl_3_ extract from the root bark of *P. sepium*. During the same period, periplosides A–C were isolated by Hikino and co-workers [32]. Additionally, periplosides A and C are equipped with an orthoester group. Later, in 2008, to study the antirheumatoid arthritis effect of the genus *Periploca*, Zhao’s group determined perperoxides A–E [33]. From further work performed by Zhao’s group, it is worth noting that the peroxy function of the sugar chains of the above thirteen pregnane glycosides (periplocosides A–F, J, K and perperoxides A–E) was revised to an orthoester group according to 2D NMR spectroscopic analysis, chemical transformation, and X-ray diffraction analysis. Hence, the names of the fourteen pregnane glycosides were assigned as periplosides A–N (**47**–**60**) in 2011. The relative configurations at the spiro-quaternary carbons of the periplosides were revised according to the single-crystal X-ray structure of periploside F (**52**) [34,35]. With further research, in 2017, the absolute configuration of periploside C (**49**) was successfully established by single-crystal X-ray diffraction analysis. In addition, nine new spiro-orthoester group-containing pregnane-type steroidal glycosides, periplosides O–V, along with 3-*O*-formyl-periploside A (**61**–**69**), were isolated from the root bark of *P. sepium* [38]. To explore more about the structure-immunosuppressive activity relationship of spiroorthoester group-containing pregnanes, Zhao and co-workers performed a chemical analysis of *P. chrysantha*. Four new periplosides were obtained, and they were named periplosides W–Y and 3-*O*-formyl-periploside F (**70**–**73**) [39].

In addition to the abovementioned twenty-seven periplosides, there are some pregnane glycosides (**74**–**86**) which have three oxygen-containing substituents in the *β* configuration at C-3 and C-17 and in the *S* configuration at C-20. In 1988, Δ^5^-pregnene-3*β*,17*α*,20(*S*)-triol (**74**) was found from the acid hydrolysates of periplocosides D, E, L–N (periplocosides L–N, **76**–**78**) [31]. However, Δ^5^-pregnene-3*β*,17*α*,20*α*-triol is an analogue of **74** without information on the absolute configuration at C-20 [73,74]. In 1987, Itokawa’s group [36] isolated a new compound S-2A, named periplocogenin (**75**). It is confusing that periplocoside M (**77**) [43] and periploside B (**48**) [44], which have two different names, have an identical chemical structure. Periplocoside N (**78**) has the same relative configuration as that of glycoside E [75] discovered in 1972. Periplocoside O (**79**) was isolated along with periplocosides J, K and F [37]. Periplocoside X (**80**) was confirmed to contain a 3,7-dioxy-heptulose group [46] according to its NMR spectroscopic data and by referring to periplocoside A. In Wu and co-workers’ search for insecticidal compounds from *P. supium*, they discovered two new glycosides, namely periplocoside P (**81**) and periplocoside NW (**82**) [47,48]. Compounds **83**–**86** are all newly found pregnane glycosides in recent ten years [18,40,43] which are different from **47**–**82** in terms of the length and constitution of the saccharide chain at C-3 or C-20.

Compounds **87**–**96** merely have two oxygenated patterns at C-3 and C-20. In 1988, Takeya and co-workers reported that compounds **88** and **89**, which were hydrolyzed with acid to yield the same aglycone (**87**) [49], were two new pregnane glycosides from the antitumor fraction of *P. sepium*. Additionally, compounds **88** and **89** both yielded **90** upon partial acid hydrolysis. Glycosides H_1_ and K, isolated in 1972 and 1969, respectively [14,76], have identical relative configurations to those of compounds **89** and **90**. Additionally, glycoside K is the first gregnane-type glycoside whose sugar chain links to the hydroxyl group, rather than C-3 of the aglycone. The biondianosides C,D (**91**,**92**), firstly isolated from *Biondia* *hemsleyana* (Warb.) Tsiang belonging to the same family [77], were also obtained from the genus *Periploca* in 2009 [52]. Periseosides C and D (**93**,**94**) are two new compounds [40]. Compound **96** [31] was established to have an *R* configuration at the C-20 position, which distinguishes it from compounds **87**–**95**.

Compounds **97**–**106** are characterized by the inclusion of two hydroxyl groups at C-16 and C-20 of the aglycones, and the configuration of C-16 was deduced to be *α*. Glycoside H_2_ (**97**) was found by Sakuma and co-workers [53] in 1980, and it showed significant potentiation of the effect of nerve growth factor. The hydrolysis of compound **97** yielded glycoside **98**. Due to a hydroxyl group at the D-ring, plocoside B (**100**) was found to exhibit higher differentiation-inducing activity [51]. Periseoside E [40] (**101**), along with periseosides A–D, was obtained in 2011. The study of the small branches of *P. graeca* collected at Marina di Vecchiano, Italy resulted in five new pregnane glycosides (**102**–**106**). It is worth mentioning that **102**–**105** are characterized by a 6-sulfated-*β*-d-glucopyranosyl unit at C-16, and the occurrence of sulfated glycosides as pregnane derivatives is an unusual finding [50]. Compound **106** bears a hydroxyl group in the *β* configuration at C-16. Compound **107** [29] is similar to **106**, with the difference between them being in the structures of the oligosaccharide moieties. Compounds **108**–**110** are equipped with the *R* configuration at C-20 and a *β*-oriented hydroxyl group at C-16.

In the 1980s, chemical investigations of *P. calophylla* led to some pregnane glycosides with a hydroxyl group at C-14 (**111**–**115**). Unfortunately, the position of the sugar moiety in calocin (**111**) was not confirmed based on spectroscopic analysis in 1982 [54]. A new pregnane ester diglycoside and a new pregnane ester aglycone (**112**,**113**) [55]—each characterized by two hydroxyl groups that are benzoylated at C-11 and C-12—both contain the structure of 12,20-di-*O*-dibenzoyl drevogenin-d. Locin (**114**) [56] displays two *β*-oriented hydroxyl groups at C-12 and C-14. Calocinin (**115**) [57] has a special 2,6-dideoxy-l-fucose sugar unit, which was determined by spectroscopic analysis and a chemical reaction. Twenty years later, a new pregnane glycoside (**116**) with a *β*-oriented hydroxyl group at C-14 was discovered from the small branches of *P. graeca* [50].

Six C_21_ steroidal glycosides (**117**–**122**) have the chemical framework of 21-methoxypregnane-20-one and feature a *β* configuration hydroxyl group at C-14, five of which are new compounds. Ye and co-workers [44] found that compound **117** was biogenetically connected to (3*β*,5*β*,14*β*)-3,14,21-trihydroxypregnan-20-one, which is a precursor of cardenolides. In addition, the presence of compound **117** could indicate the pregnane pathway for the biosynthesis of cardenolides in *P. sepium*. Perisepiumoside E (**118**) [58], a glycoside of **117**, was isolated in 2008. Two years later, a new pregnane glycoside (**119**) with an *α-*oriented hydroxyl group at C-17, together with its known pregnane genin (**120**), was obtained [18]. In comparison with compound **120**, the pregnane glycosides **121** and **122** [43] contain disparate saccharide chains.

It is noteworthy that the carbon side-chain at C-17 of the ten distinct compounds (**123**–**132**) is *α-*oriented. Interestingly, these compounds all possess a *β*-oriented hydroxyl group at C-14, and eight are new compounds. An aglycon calogenin (**123**) [59] was established as containing a C-17 hydroxyethyl chain in *α* configuration. However, it is puzzling that the *β* configuration at C-17 was assigned to calogenin 3-*O*-*β*-d-digitalopyranoside-20-*O*-*β*-d-canaropyranoside (**116**) [50]. Plocinine (**124**) [60] is characterized by two cinnamoyl groups assigned to the methine protons at C-12 and C-20 of ornogenin. Compounds **125**–**128** are a series of 21-methoxypregnanes with a hydroxyl group in the *β* configuration at C-17. Similar to compounds **117**–**122**, the pregnane glycosides **129**–**132** are also 21-methoxypregnane-20-one derivatives, but the remarkable difference is in the configurations at C-17. Furthermore, compounds **129** and **132** are stereoisomers at C-17 of **120** and **119**, respectively. What is concerning is that compound **81** [47] was assigned the trivial name of periplocoside P in 2014, whereas **132** [61] was given the same name in 2015.

The vast majority of C_21_ steroid constituents from genus *Periploca* have one unsaturated double bond at C-5. Nevertheless, compounds **133**–**138**, in particular, have double bonds at other positions, such as C-14, C-16 and C-6. In 1968, compound **134** [62] was obtained as a dehydrated product of Δ^5^-pregnene-3*β*,17*α*,20*α*-triol diacetate. Neridienone A (**135**) has three olefinic bonds [41]. In a search for compounds with antioxidant and melanogenesis-inhibitory abilities from *P. forrestii*, three known pregnane glycosides (**136**–**138**) [63] bearing an ethylene bond at C-6 were discovered.

#### 3.1.3. Phytosterols

Four usual phytosterols (**139**–**142**, Table 2, Figure 4) were obtained from the genus *Periploca*.

### 3.2. Carbohydrates

A total of twenty-six carbohydrates have been isolated from the genus *Periploca* and mainly came from *P. sepium* and *P. forrestii*, eighteen of which are new compounds. Compounds **143**–**145** are monosaccharides, and compounds **146**–**168** are oligosaccharides. Carbohydrates can be considered characteristic components of *Periploca* species because most of them contain one or more 2,6-dideoxysugars or/and 6-deoxysugars and their derivatives. What is noticeable is that the sugar sequences of these oligosaccharides are ruled by the regularity in cardiac- and pregnane-type glycosides of the Apocynaceae (Asclepiadaceae) plants [78].

**Figure 5:**
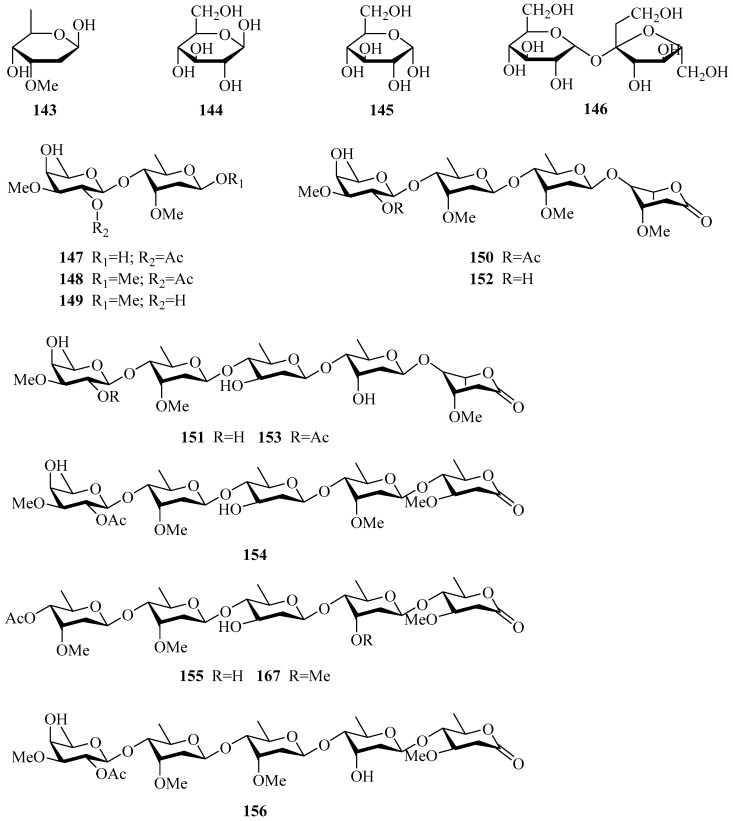

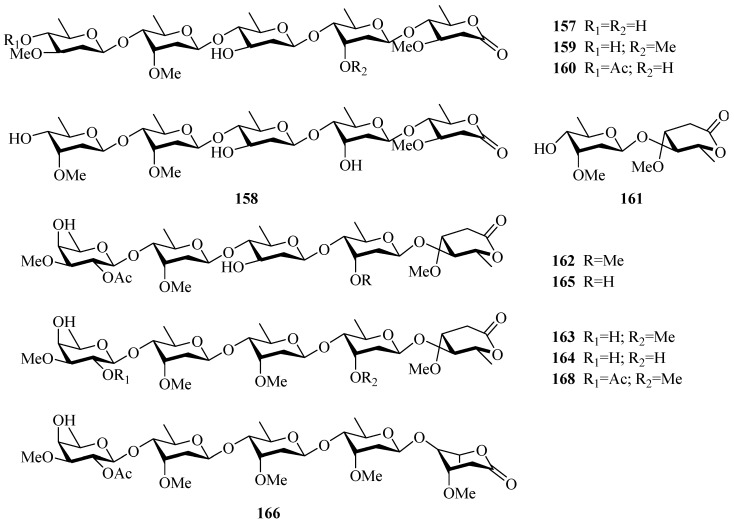
Carbohydrates from the genus *Periploca.*

The oligosaccharides (**147**,**148**) were hydrolysis products of glycosides from *P. sepium.* Similarly, upon partial acid hydrolysis, compound **88** yielded **149**, a deacetylation product of **147**. Compounds **150**–**153** were isolated and elucidated as four new-type oligosaccharides in 1977, which were the first examples of oligosaccharides composed of 2,6-dideoxyaldonic lactone and 2,6-dideoxysugars [78]. Thirty years later, Zhao’s group [33] engaged in the discovery of new immunosuppressive compounds from traditional Chinese medicine, which resulted in five oligosaccharides being obtained. Among them, three were new compounds, named perisaccharides A–C (**154**–**156**). In their ongoing chemical investigation of *P. forrestii*, four new oligosaccharides (**157**–**160**) [29], characterized by diverse sugar units and an oleandro-1,5-lactone, were elucidated. In 2010, Ye and co-workers [79] reported five new oligosaccharides, perisesaccharides A–E (**161**–**165**), and assigned their absolute configurations. Intriguingly, they put forward that the conformation of the oleandronic acid *δ*-lactone should be confirmed as “boat,” rather than the “chair” assessment in previous literatures, on the basis of a combination of X-ray diffraction analysis, a modified Mosher’s method, CD measurements and acid hydrolysis. Additionally, the “boat” conformation of oleandronic acid *δ*-lactone was reported for the first time. Perisesaccharide F (**166**) [80], as a new natural product, was isolated from *P. sepium*, while another new oligosaccharide (**167**) [81] was discovered from *P. calophylla.* The structures, names, corresponding sources, parts of plants and references of these compounds are summarized in Table 3 and Figure 5. A small number of polysaccharides isolated from this genus are not listed here.

### 3.3. Terpenoids

**Figure 6:**
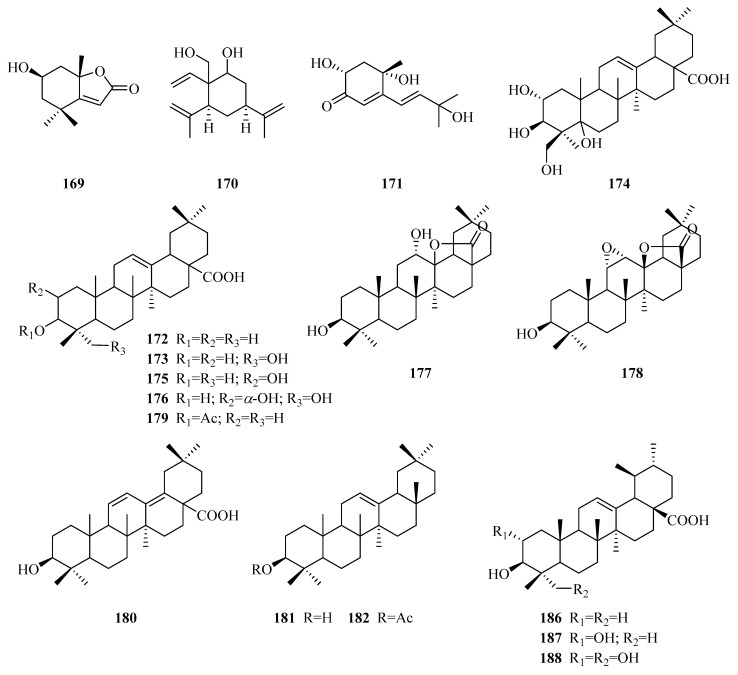

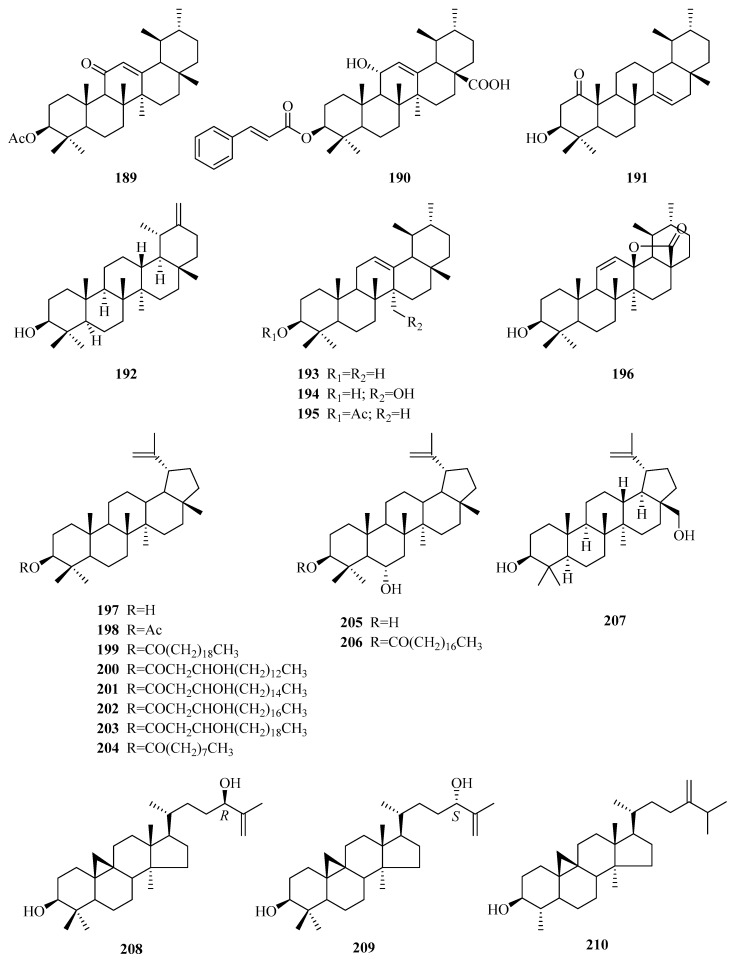
Terpenoids from the genus *Periploca*.

There are forty-two terpenoids (**169**–**210**) composed of triterpenoids and three other types of terpenoids (**169**–**171**). According to their structural characteristics, these triterpenoids (**172**–**210**) are divided into four sub-types, namely oleanane-type triterpenes, ursane-type triterpenes, lupane-type triterpenes and cycloartane-type triterpenes. Their structures, names, corresponding sources, parts of plants and references are illuminated in Table 4 and Figure 6.

Loliolide (**169**) [73] is a monoterpene lactone contained in *P. forrestii*. Periplocadiol (**170**) is a new elemane-type sesquitone [70]. A new norterpenoid (**171**) [84] with a Δ^4^-3-one unit was found in the whole plant of *P. aphylla*.

Compounds **172**–**185** are a series of oleanane-type triterpenes. In 1971, oleanolic acid (**172**), together with maslinic acid (**175**), was first isolated from the genus *Periploca* [70]. Two known triterpenes (**177**,**178**) [10,87] characterized by a 13,28-lactone ring were discovered from *P. laevigata* and *P. somaliensis*, respectively. Additionally, three new triterpenoid acids of the oleanane series, named P1, P2, P3 (**183**–**185**), were isolated from the twigs of *P. calophylla*, without definite chemical structures [89]. Eleven ursane-type triterpenes (**186**–**196**) were found in the species of genus *Periploca*. Ursolic acid (**186**) [94] was acquired in 1987 from *P. nigrescens*. Moreover, these triterpenes also illuminated a new triterpene (**196**), which also has a 13,28-lactone ring [94]. Compounds **197**–**207** are triterpenes of the lupane type, and seven (**197**–**203**) of them were obtained from the latex of *P. laevigata* collected in Tunisia [87]. In addition, compounds **199**–**204** have the characteristic of a long-chain alkanonic ester or a *β*-hydroxy fatty acid ester at C-3. The structures of two new triterpenes (**205**,**206**) from *P. aphylla* resembled each other very closely, but the remarkable difference was in the substituent at C-3. Due to the difference, compound **205** exhibited strong inhibition of *α*-glucosidase type VI and a temperate antibacterial activity, while **206** was inactive or weak [67]. There are three cycloartane-type triterpenes (**208**–**210**) [92] with a hydroxyl group in the *β* configuration at C-3 which were isolated from this genus for the first time.

### 3.4. Phenylpropanoids

**Figure 7:**
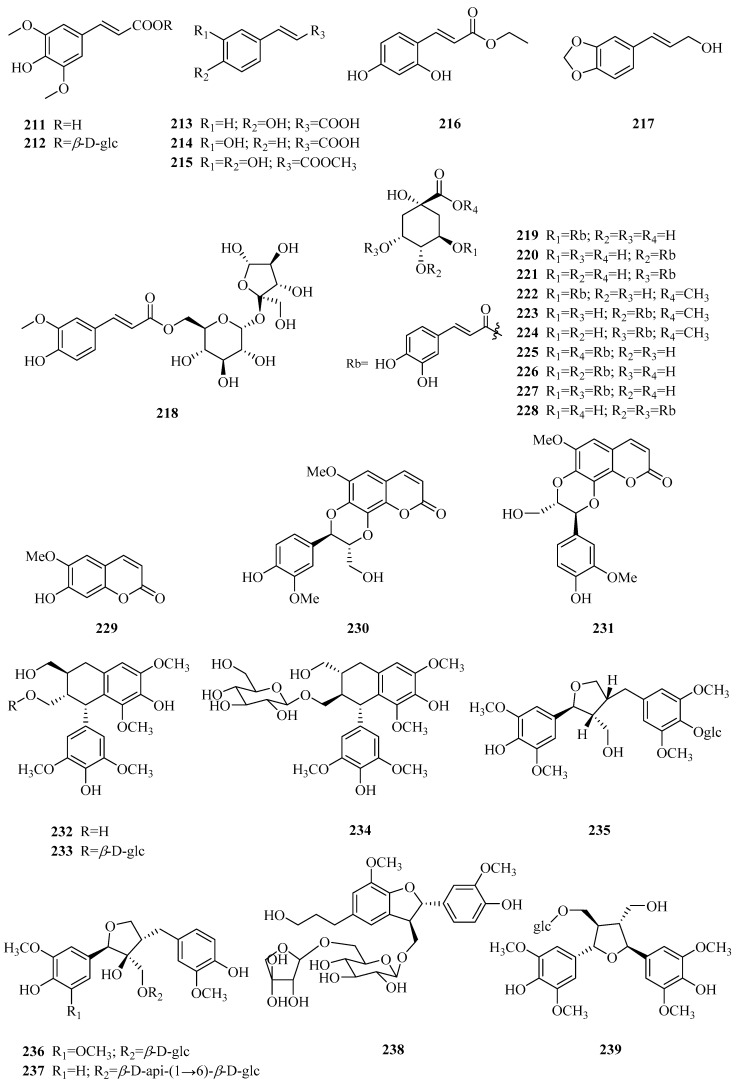

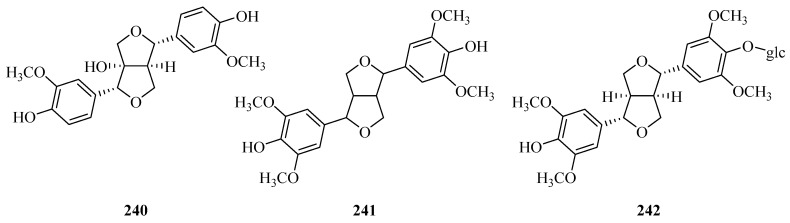
Phenylpropanoids from the genus *Periploca.*

To date, thirty-two phenylpropanoids (**211**–**242**) have been reported, and they are divided into three categories: Simple phenylpropanoids, coumarins, and lignans. Their structures, names, corresponding sources, parts of plants and references are illuminated in Table 5 and Figure 7. Compounds **211**–**228** are simple phenylpropanoid compounds, among which compounds **219**–**228** [74] are a series of caffeoylquinic acids which were isolated from the hydrosoluble fraction of *P. forrestii* for the first time in 2017. Scopoletin and cleomiscosins A and B (**229**–**231**) are the only three coumarins from *Periploca* species [66,97]. In addition, there are eleven lignans (**232**–**242**). (+)-lyoniresinol (**232**) is an arylnaphthalene-type lignin [84], while tortoside B (**235**) is a tetrahydrofurane-type lignan [98]. Two new tetrahydrofurane-type lignins (**236**,**237**), along with four known lignans (**233**,**234**,**238**,**239**), were determined from *P. forrestii* in 2017 [63]. In addition, the remaining three compounds **240**–**242** appear as furoferran-type lignins [8,73,99].

### 3.5. Flavonoids

**Figure 8:**
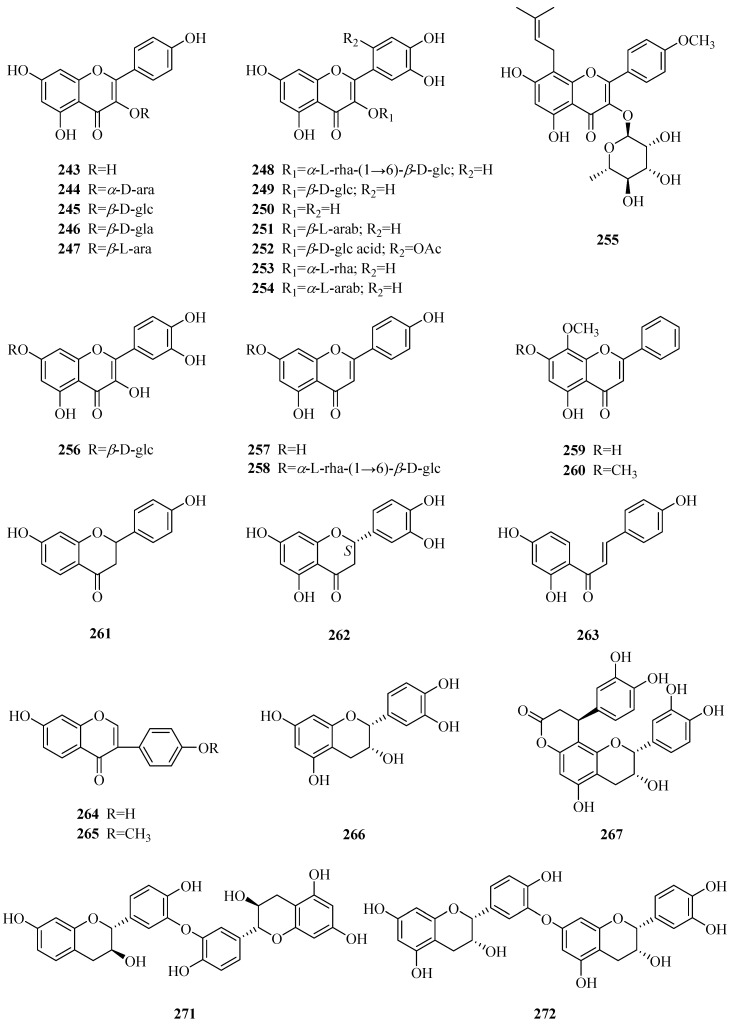

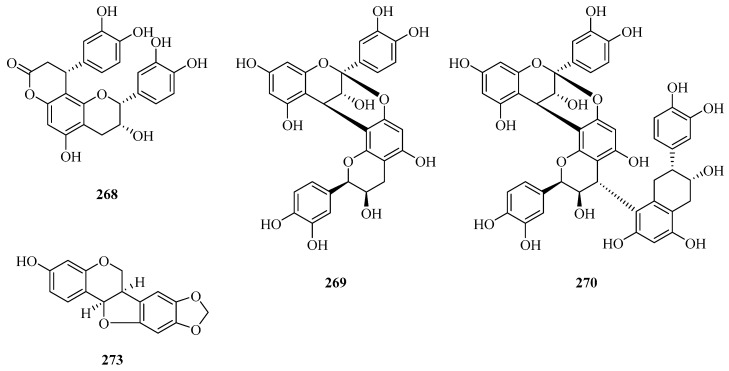
Flavonoids from the genus *Periploca*.

A great variety of flavonoids (**243**–**273**, Table 6, Figure 8) have been isolated and identified from this genus, and most were obtained in the past fifteen years. Familiar flavonols, such as kaempferol, quercetin and their derivatives (**243**–**256**), are also found in *Periploca* species. Compounds **257**–**260** are four simple flavones, while **261** and **262** are two common flavanones. There are three usual chalcones and isoflavones (**263**–**265**). The genus *Periploca* is also characterized by containing flavanes (**266**–**272**) [63,84]. Moreover, (−)-maackiain (**273**) is a pterocarpan [45].

### 3.6. Quinones

**Figure 9:**
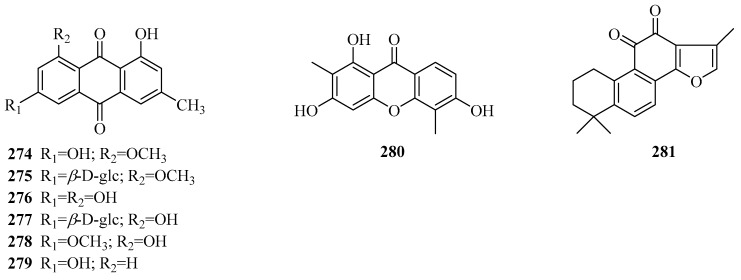
Quinones from the genus *Periploca*.

A total of eight quinone compounds (Table 7, Figure 9) has been found in the genus *Periploca*, and seven of them were derived from *P. forrestii*. Compounds **274**–**279** are six emodin-type anthraquinones. An anthrone (**280**) was reported for the first time from *P.* *aphylla* [84]. Additionally, tanshinone IIA (**281**) [111] possesses a diterpenoid quinone skeleton, which was discovered from the portion of *P. forrestii* with anti-inflammatory activity.

### 3.7. Aromatics

**Figure 10:**
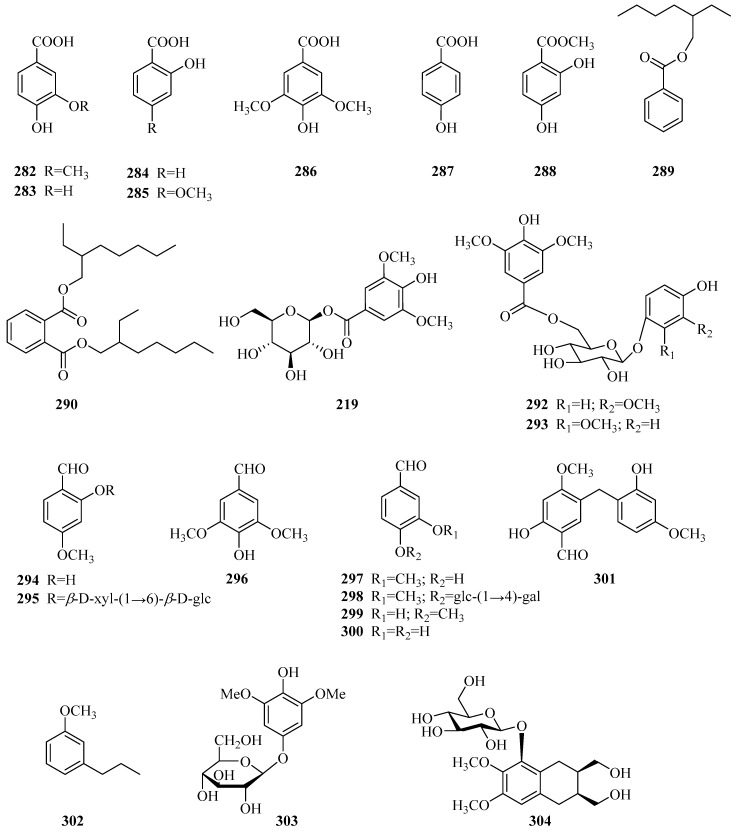
Aromatics from the genus *Periploca*.

Twelve aromatic acids and their derivatives **282**–**293** (Table 8, Figure 10) were discovered from the genus *Periploca.* These carboxylic acid groups could be esterified with alkanols or form ester glycosides with sugar moieties. Aromatics **294**–**301** are eight aromatic aldehydes and their derivatives. 4-methoxy salicylaldehyde (**294**) was first reported in the bark of *P. graeca* in 1935 [113]. Compound **301** is a new diphenylmethane [44]. Compounds **302** and **303** are two aromatic ether compounds, and a new naphthalene derivative named periplocain A (**304**) [108] was discovered in 2016.

### 3.8. Others

**Figure 11:**
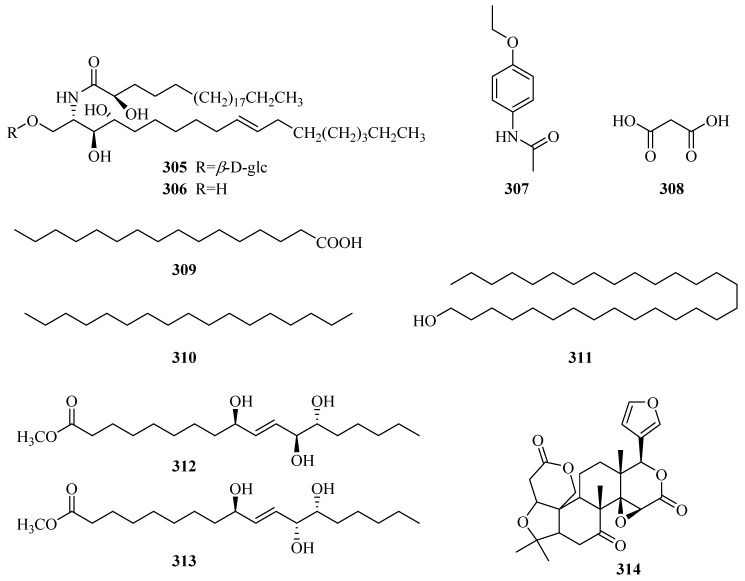
Other compounds from the genus *Periploca*.

Two ceramide compounds **305** and **306** were obtained from the trichloromethane fraction of the 80% alcohol extract of *P. forrestii* for the first time in 2014 [116]. There are six aliphatic compounds (**308**–**313**) and two stereoisomeric fatty esters (**312**,**313**) [63]. Obaculactone (**314**) is a lactone [80]. Their structures, names, corresponding sources, parts of plants and references are showed in Table 9 and Figure 11. Additionally, it should be noted that some researchers have reported that the plants of genus *Periploca* are rich in volatile oils, whereas the volatile oil components detected online are not listed in detail in this paper.

## 4. Biological Activities 

The extracts of the genus *Periploca* have been investigated for a series of biological activities since the 1920s. It has been found that they show diverse and valuable activities, including cardiotonic, anti-inflammatory, immunosuppressive, antitumor, antimicrobial, antioxidant, insecticidal and other properties. The biological activities of the genus *Periploca*, primarily interrelated species and active components are illustrated in Table 10.

### 4.1. Cardiotonic Effect

As early as the 1920s, periplocin (**1**) was detected to show digitalis-like cardiotonic capacity. Periplocin enhanced the tonus and the contractility of the cardiac muscle, as well as the tonus of the arterial muscle, whereas it led to cardiac irregularity and systolic arrest of the rat heart at excessive doses [117]. Additionally, a 10% infusion gave rise to a decrease of the frequency of the heartbeat and an increase of the tonus, arterial pressure and diuresis [118]. Through its effects on frog and rabbit hearts in situ and isolated guinea pig hearts, the total glycosides fraction extracted from the fresh stem bark of *P. forrestii* displayed that its cardiotonic property was analogous to that of g-strophanthin, and the average lethal dose in pigeons was 5.9 ± 1.0 mg/kg. Concerning the changes of rabbit electrocardiograms, both compounds produced the phenomena of prolonged R-R and P-R intervals, a shortened QRS interval and a flattened T wave [119]. The ethanol extract of *P. calophylla*, with an average lethal dose in pigeons of 31.86 ± 1.62 mg/kg, was also proven to be equipped with cardiotonic function via experiments on frog and rabbit hearts in situ, isolated guinea pig hearts, and through electrocardiograms in cats [120]. Wang’s group discovered that periplocin could improve the left ventricular structure and function in rats with chronic heart failure at a dose of 8 mg/kg [121]. They inferred that the underlying mechanism of periplocin against heart failure might involve its ability to increase the expression of sarco endoplasmic reticulum Ca^2+^-ATPase mRNA, decrease the expression of phospholamban mRNA and improve the value of PLB/SERCA in chronic heart failure-model rats [122] The proliferative activity of periplocin was verified in mouse cardiac microvascular endothelial cells by Gao and co-workers [123]. Compared with periplocin (**1**), periplogenin (**2**) and xysmalogenin (**25**) both showed positive inotropic and negative chronotropic effects on isolated rat hearts, but their activities were weaker and failed more quickly [124].

### 4.2. Anti-Inflammatory and Immunosuppressive Effects

Pharmacological tests determined that the ethanol extracts, volatile oils and water extracts mainly containing polysaccharides of *P. forrestii* all had anti-acute inflammation properties [125,126,127]. The ethanol extract of *P. forrestii* displayed anti-inflammatory activity which was connected to reducing the protein expression of inducible nitric oxide synthase and cyclooxygenase-2 (COX-2); to the production of inflammation factors, including tumor necrosis factor (TNF)-*α*, interleukin (IL)-6, nitric oxide and prostaglandin E_2_ (PGE_2_); and, mainly, to the suppression of lipopolysaccharide-mediated stimulation of the nuclear factor-*κ*B (NF-*κ*B) and mitogen-activated protein kinases signaling pathways [128]. Additionally, the extract of *P. laevigata* was also able to ameliorate carrageenan-induced edema and acetic acid-induced abdominal writhing, in addition to enhancing the latency in a hot plate test [129]. In 1997, three triterpenes from *P. sepium*, named *α*-amyrin (**193**), *α*-amyrin acetate (**195**), and *β*-amyrin acetate (**182**), were shown to have anti-inflammatory effects in animal experiments [130]. At a dose of 20 *μ*g/mL, Periplocin (**1**) could distinctly inhibit histamine release from mast cells cultured in vitro and from mast cells of sensitized rats. As such, periplocin was considered to be one of the effective anti-inflammatory ingredients of *P. sepium* by Gu’s group [131]. It is worth noting that periplocoside A (**49**) showed a sufficiently preventative effect on concanavaline A-induced hepatitis by inhibiting natural killer T-derived inflammatory cytokine production, which suggested that periplocoside A possesses therapeutic potential for the treatment of human autoimmune-related hepatitis [132].

In 2005, periplocoside E (**47**) was first found to specifically and markedly inhibit the activation of extracellular signal-regulated kinase and Jun N-terminal kinase, while the activation of p38 was not influenced by T cells stimulated with anti-CD3, showing its potency for the treatment of T cell-mediated disorders [133]. In addition, its immunosuppressive efficacy was confirmed again by the effect of treatment with periploside A (**47**) on a positive selection of thymocytes from C57BL/6 mice in vitro [134]. After finding that the crude extract of *P. sepium* and periplocoside E had the ability to inhibit the proliferation of T cells, Zhao’s group carried out a systematic chemical investigation and searched for immunosuppressive compounds, making vital contributions to this domain. Compared with the immunosuppressants rapamycin (IC_50_ 0.19 *μ*M) and cyclosporin A (IC_50_ 0.27 *μ*M), nine pregnane glycosides (**47**,**49**–**55**,**58**) characterized by an orthoester group displayed significant inhibitory activities against the proliferation of T lymphocytes in vitro, with IC_50_ values ranging from 0.29 to 1.97 *μ*M. However, other pregnane glycosides without the orthoester group showed no apparent inhibitory activity [33]. Twelve spiro-orthoester group-containing periplosides (**49**,**59**–**69**) were evaluated for their inhibitory activities. Among them, periploside C (**49**), the most abundant spiro-orthoester moiety-bearing component in the root bark of *P. sepium*, exhibited the best selective index (SI) value at 82.5. More notable is that the length and constitution of the saccharide chain in this compound class have a crucial impact on both the suppressive activity and the SI value. Nevertheless, the substitution of a formyl group in replacement of the 4,6-dideoxy-3-*O*-methyl-Δ^3^-2-hexosulose function at the C-3 position of the aglycone might not affect this activity in periplosides [38]. Additionally, Zhao’s group demonstrated the potential mechanism of the immunosuppressive ability of periplocoside A (periploside C, **49**). Though inhibiting IL-17 production and suppressing the differentiation of Th17 cells in vitro, the oral administration of periplocoside A ameliorated experimental autoimmune encephalomyelitis [135]. The other four compounds of the same type (**70**–**73**) were equipped with notable inhibitory activities against the proliferation of B and T lymphocytes, and advantageous selective index values comparable to those of cyclosporin A, which perfectly supplemented the immunosuppressive potency of the periplosides [39].

In 2006, lupane acetate (**198**) was found to induce the differentiation and maturation of dendritic cells, upregulate cytokines production, and enhance the immune activity of dendritic cells in vitro [136]. The ability of lupane acetate to increase the immune response of lymphocytes and macrophages from human peripheral blood might be associated with its ability to kill carcinoma cells [137]. Additionally, periplocin (**1**) was also discovered to protect the immune organs of tumor-bearing mice, obviously enhance the lymphocyte proliferation in mice spleens, and stimulate the production of TNF-*α*, IL-2 and IL-12, which indicated that it had immunoregulatory effects [138]. The polysaccharide (HGT-5A) from *P. forrestii* showed immunosuppressive effects on the cellular immune response and that its four ingredients played a major role [139]. However, three oligosaccharides (**154**–**156**) displayed no activity against the proliferation of T lymphocytes in vitro [33]. In 2017, by in vitro experimentation, suppressing hyaluronidase, microwave extraction combined ultrasonic pretreatment of flavonoids from *P. forrestii* were found to produce resistance to allergies with an IC_50_ value of 1.033 mg/mL [140].

Rheumatoid arthritis (RA) is a chronic autoimmune inflammatory disease, and extracts of *P. forrestii* and *P. sepium* showed favorable anti-arthritis efficacies. In 2004, the aqueous extract of *P. sepium* was found to inhibit the growth and IL-6 production of human rheumatoid arthritis-derived fibroblast-like cells in a dose-dependent manner [141]. The water-soluble components of *P. forrestii* could suppress the inflammatory response in collagen-induced arthritic rats through regulating the expression of the TAK-1, TAB-1, NF-*κ*B p65, MCP-1 and CXCL-1 proteins [142]. Huang’s group [143] revealed that a 60% ethanol extract of *P. forrestii* possessed activity against RA capacity. They inferred that the potential mechanism might be associated with the regulation of immune organ function and the levels of pro-inflammatory cytokines containing IL-1*β*, IL-6 and TNF-*α*. Liang and co-workers [144] drew a conclusion that the effect of a 50% ethanol extract of *P. forrestii* on RA was concerned not only with restraining the proliferation of rheumatoid arthritis synovial fibroblasts but also with the downregulation of the expression of COX-2 and PGE_2_. Through further investigation, Feng’s group [145] provided new insight into the underlying mechanism of the therapeutic action in collagen-induced arthritis (CIA). By inhibiting the activation of Src kinase and the nuclear translocation of NF-*κ*B in rats, the ethanol extract of *P. forrestii* was shown to be a potential candidate for the treatment of patients with RA. The anti-arthritis effect of *P. forrestii* was discovered to be positively correlated with the contents of total flavonoids and total saponins, and the influence of the total saponins was greater than that of the total flavonoids [146]. Sun’s group [147] verified that *P. forrestii* saponin and periplocin (**1**) performed prophylactic therapeutic action against autoimmune arthritis by controlling the systemic autoimmune responses and local inflammation and bone destruction of the joints. Additionally, they deduced that the protective mechanism of *P. forrestii* saponin toward CIA had something to do with regulatory actions on proinflammatory factors, as well as on the crosstalk between NF-*κ*B and c-Fos/AP-1 in vivo and in vitro [148]. Another recent biological study of the cardenolide-rich and caffeoylquinic acid-rich fractions of *P. forrestii* demonstrated that the inhibitory activities toward the NF-*κ*B and MAPK signaling pathways might be involved in the antiarthritic effect based on a set of experimental results [149].

### 4.3. Antitumor Ability

Abundant experiments concerning the antitumor activities of *P. sepium* were carried out by Shan and co-workers. Above all, Shan’s group explored the role of periplocin (**1**) in affecting human cancer cell lines in vitro from 2005 to 2017. They found that periplocin expressed a noteworthy inhibitory effect on diverse cancer cell lines—including hepatocellular carcinoma cells, colon carcinoma cells, lung carcinoma cells, and breast carcinoma cells—basically though blocking the cell cycle and inducing apoptosis [150,151,152,153]. Furthermore, the potential antitumor mechanisms of periplocin were inferred by Shan’s group. Periplocin was detected to suppress the proliferation and induce apoptosis of the human hepatocarcinoma cell line SMMC-7721 via restraining Stat3 signal transduction [150]. By the downregulation of the Wnt/*β*-catenin signaling pathway, periplocin (0.5 *μ*g/mL) markedly inhibited proliferation of the human colon carcinoma cell line SW840 [151]. The probable mechanism by which periplocin induced apoptosis in the human lung cancer cell line A549 was related to the downregulation of the survivin mRNA and protein [152]. In addition, periplocin had an effect on breast carcinoma MCF-7 cells with the IC_50_ value of 4.88 ± 0.16 ng/mL, which was associated with the increased expression of the mRNA and protein of gene p21^WAF1/CIP1^ [153]. Additionally, periplocin (**1**) also displayed the ability to suppress the growth of human (A549) and mouse (LL/2) lung cancer cells in vitro and in vivo by blocking the protein kinase B/extracellular signal regulated kinase signal pathway [154]. In 2013, Pan’s group deduced that periplocin (**1**) sensitized TNF-related apoptosis-inducing ligand-resistant human hepatocellular carcinoma cells via inducing the expression of the death receptors 4 and fas-associated death domain and the downregulation several inhibitors of apoptosis, which resulted in the activation of caspases 3, 8, and 9 and led to cell apoptosis [155]. Moreover, due to the inducing of DNA double strand breaks and death-receptor mediated apoptosis in liposarcoma cells, periplocin (**1**) was considered to be a promising lead compound for the development of new sarcoma therapeutics by Bauer’s group [156].

Additionally, periforoside E (**27**) showed higher cytotoxicity toward the A549 line than toward other lines. Notable is that 3*β*,5*β*-dihydroxy-14-en-card-20(22)-enolide (**26**) and periforoside D (**45**) were both inactive in terms of cytotoxic activity, indicating that the 14-hydroxy group and the C/D ring junction were responsible for this effect [19]. Moreover, the three 17*α*-cardiotonic steroids (**38**-**40**) showed apparently higher IC_50_ values (5.4 and 7.3 *μ*M) in vitro in the hormone-independent prostate cancer cell line PC3 than those of 17*β*-isomers. Additionally, in the 17α-cardenolides, the presence of sugar units of these cardenolides had nothing to do with the IC_50_ values. However, a fascinating series of 17*β*-cardiotonic steroids (**1,3**–**5,24**), with a 14*β* hydroxyl group and at least one sugar molecule, demonstrated strong antiproliferative effects with IC_50_ values between 18 and 50 nM [15]. Four cardenolides (**1**–**3**,**38**) were tested in the human monocytic leukemia U937 and androgen-independent prostate adenocarcinoma PC3 cell lines. Periplocin (**1**) and periplocymarin (**3**) showed more effective activity toward tumor cells due to the activation of apoptotic pathways in PC3 cells and in U937 cells due to the induction of cell cycle impairment [157]. More interesting is that periplocymarin (**3**) was unlikely to encounter drug–drug interactions with P-glycoprotein and cytochrome P450s, suggesting that it should be taken forward for further investigations in drug development [158].

In addition to cardenolides, other types of compounds have been examined for their cytotoxic activities. Periseosides C **(93)** showed moderate inhibitory activity against the ConA-stimulated splenocyte proliferation in vitro [40]. Two C_21_ pregnane-type steroids (**52**,**77**) and four known oligosaccharides (**168**,**155**,**162**,**163**) were tested for their cytotoxicities against the A-549 and HepG2 human cancer cell lines with IC_50_ values ranging from 0.61 to 7.68 *μ*M by Wang’s group [43]. Interestingly, compound **168**, a derivative of **163** with the acetyl group at C-2 of the *β*-D-digitalopyranosyl moiety, exhibited moderate activities, while **163** and others were not active, which implied that the presence of an acetyl group might be related to the cytotoxic activity. Besides, Shan’s group discovered that baohuoside-I (**255**) restrained the proliferation of and arrested Eca109 human esophageal squamous carcinoma cell cycles, which might be associated with the downregulation of Cyclin B1 mRNA expression [159]. As the research continued, they reported that baohuoside-I (**255**), by inhibiting *β*-catenin-dependent signaling pathways, significantly inhibited the proliferation of the cells in a time- and dose-dependent manner with the IC_50_ value of 24.8 *μ*g/mL at 48 h and induced apoptosis of Eca109 cells in vitro and in vivo [160]. Coincidentally, lupeal acetate (**198**) was also found to reduce the incidence of N-nitrosomethylbenzylamine-induced esophageal tumors from 93.3% to 33.3% in rats after twenty-five weeks of treatment, which might have occurred through the activation of glycogen synthase kinase-3*β* expression and the inhibition of *β*-catenin and c-myc expression [161].

To search for potent compounds against carcinoma disease, various extracts of the genus *Periploca* have been studied continually. In the 1980s, Japanese scholars were the first to discover that the methanol extract of *P. sepium* was equipped with significant anticancer activity toward sarcoma 180 ascites in mice with ascites cancer [36,41]. The ethanol extract of *P. sepium* had effects on the proliferation and apoptosis of human esophageal carcinoma cells, and this action might be related to the upregulation of Rb protein expression and downregulation of the expression of apoptosis suppressor genes and the induction of apoptosis [162,163]. Additionally, the ethanol extract of *P. sepium* was suspended in water and extracted by ethyl acetate, and the ethyl acetate extract was capable of inducing apoptosis in human esophageal carcinoma TE-13 cells because of the downregulation of cyclin-dependent kinase 4 expression [164]. Analogously, the ethanol extract of *P. forrestii* stems was suspended in water and extracted sequentially with four different polar solvents. Among them, ethyl acetate extract emerged as having the strongest inhibitory property against the proliferation of K562 tumor cells, with the maximum inhibitory of 80. 71% at 25 mg/L, from which periplocin was isolated [165]. In 1995, Umehara and co-workers [51] suspended the methanol extract of *P. sepium* in water and extracted it with ethyl acetate. The water layer displayed differentiation-inducing activity against mouse myeloid leukemia cells. Later, the effect of the water extract of *P. sepium* on the differentiation of K562 tumor cells was detected, which showed that this extract could kill K562 cells but could not induce differentiation [166]. The water extract of *P. sepium* also inhibited the growth of gastric carcinoma BGC-823 cells and induced apoptosis, both of which which was associated with the downregulation of the expression of B-cell lymphoma-2 and surviving genes and proteins and to the upregulation of the expression of bax genes and proteins [167].

In brief, the complex mixtures and some pure chemical components from the plants of genus *Periploca* have presented remarkable cytotoxicity activities toward about ten different carcinoma cells, while a large proportion of investigations have focused on cytotoxic assays in vitro. For the sake of discovering antitumor lead compounds, there are still many studies worth further development.

### 4.4. Antimicrobial and Antioxidant Abilities

Triterpenes from this genus have been known to exhibit antibacterial properties since 2000. 3*β*,6*α*-dihydroxylup-20(29)-ene (**205**) showed moderate activity against *Staphylococcus pyogenes* [67]. Based on the antibacterial activities of oleanolic acid (**172**), maslinic acid acetate, *β*-amyrin (**181**) and their derivatives, Jegham’s group [168] speculated the antibacterial structure-activity relationship of triterpenes. The free *β*-hydroxyl group at C-3 and the *β*-oriented carboxyl function at C-17 were closely associated with the antibacterial activities. Interestingly, the amine linked to the triterpenoid skeleton contributed to the activity against *Pseudomonas aeruginosa*, one of the bacteria most resistant to antibiotics and antiseptics.

The petroleum ether extract with the highest content of palmitic acid (**309**) from *P. forrestii* was detected to have antimicrobial properties [169]. Due to containing relatively high proportions of aldehydes and oxygenated monoterpenes, the essential oil from *P. laevigata* root bark was equipped with significant antibacterial and antifungal activities [170]. Larhsini and co-workers [171] found that the essential oil from *P. laevigata* leaves showed antimicrobial and antioxidant activities and had a good synergistic effect in association with conventional antibiotics. The root bark essential oil of *P. sepium* and 4-methoxysalicylaldehyde (**294**), a main component of the oil (78.8% of the total), were both elaborated to possess antimicrobial properties and moderate antioxidant activities, which indicated that the major portion of these antimicrobial and antioxidant activities was due to the presence of the aldehyde in the oil [172]. In addition, the activities of 4-methoxysalicylaldehyde (**294**) isolated from *P. sepium* oil and its six derivatives against six foodborne bacteria were evaluated by Lee’s group [173]. Compound **294** (MIC 30.1–67.3 *μ*g/mL) and 4-hydroxysalicylaldehyde (MIC 41.1–61.5 *μ*g/mL) both inhibited the growth of the six foodborne bacteria with finer effects than did the other derivatives.

The antioxidant capacity of phenols is intimately related to their content and the number of hydroxyl groups. The methanol extract with the highest contents of phenolics and flavonoids from *P. laevigata* displayed the greatest antioxidant properties, followed by the water and ethyl acetate extracts [174]. Intriguingly, six lignins (**236**,**237**) were evaluated for their antioxidant abilities, and they showed no or minor antioxidant activities. Feng’s group illustrated the probable structure-activity relationship. The more original hydroxyl groups of phenols are replaced by methoxyl groups, the lower the antioxidant activity is [63]. They also detected that flavanes (**266**–**268**,**270**) which exerted high radical-scavenging activities with IC_50_ values ranging from 14.61 to 28.80 *μ*M. Additionally, aesculitannin B (**270**), a flavane trimer, was able to shrink *α*-melanocyte-stimulating hormone-induced melanogenesis by inhibiting the expression of microphthalmia-associated transcription factor and tyrosinase in a dose-dependent manner, owing to the multiple phenolic hydroxy groups [63]. Bisflavan-3-ols **271** and **272** from the ethyl acetate fraction of *P. aphylla* displayed evident inhibitory potential against the enzyme lipoxygenase with IC_50_ values of 19.7 and 13.5 *μ*M. Malik and co-workers speculated that the stereochemistry of **272** was probably more conducive to the inhibition of lipoxygenase enzyme than that of **271** [84].

In the most recent three years, polysaccharides acquired from this genus have also been detected to show antioxidant and antibacterial capacities. Three crude Cortex Periplocae polysaccharides (CPPs 1–3) from the root bark of *P. sepium* presented evident antioxidant activities in four assays in vitro [175]. A novel polysaccharide (PLP1) from *P. laevigata* root bark displayed antioxidant activity in a dose-dependent manner and showed strong antibacterial activity against several Gram (+) and Gram (−) strains. Hence, it was shown to be a promising source for polysaccharides in the medical and food industries [176]. The polysaccharides isolated from *P. angustifolia* (PAPS) were shown to decrease the rates of lipid peroxidation and protein glycation in vitro. Furthermore, PAPS could defend human embryonic kidney cell HEK293 against oxidative stress and renal tissue against cadmium toxicity [177].

Furthermore, the antioxidant capacity of PAPS was demonstrated by the hepatoprotective potency toward cadmium chloride (CdCl_2_)-induced toxicity in rats, and it could ameliorate the alteration of liver tissue [178]. Athmouni’s group [179] testified that the methanolic extract of *Periploca angustifolia* leaves also possessed antioxidant ability and exerted salutary effects in the treatment of Cd-induced hepatotoxicity. The methanol extract of *P. hydaspidis*, which is a less-studied species found in Pakistan, had the ability to adjust the levels of various parameters provoked by CCl_4_ toxicity in a dose-dependent manner due to the presence of antioxidant and anti-inflammatory constituents in the methanol extract [180]. What is striking is that ZnO nanostructures with tremendous potential against multidrug-resistant *E. coli*, *S. marcescens* and *E. cloacae* were synthesized through reduction by *P. aphylla* aqueous extract without the utilization of any acid or base. In this manner, the aqueous extract of *P. aphylla* was proven to be a valid chelating agent [181].

### 4.5. Insecticidal Activity

Since 2004, extracts from the plants of genus *Periploca* have been found to possess favorable insecticidal activities and promising agricultural value. The ethanol extract from the root bark of *P. sepium* had a significant repellent effect against the oviposition of imported cabbage worms [182]. Additionally, the benzene fraction from that ethanol extract showed strong antifeeding and poisoning effects on *Plutella xylostella* larvae [183]. The methanol extract of *P. aphylla* showed activity against *Leishmania infantum* (IC_50_ 6.0 *μ*g/mL, SI 4.0) [184]. The volatile oil from the root bark of *P. sepium* showed forceful contact-killing activity against *Myzus avenae*, and its main component was 4-methoxysalicylaldehyde (**294**), which also displayed antinematodal activity against *Bursaphelenchus xylophilus* at a minimum effective dose of 200 *μ*g/ball [114,185]. In 2012, it was also proven that the dominating component in the oil (**294**) was active against *Drosophila melanogaster* L. and the maize weevil (*Sitophilus zeamais*) with the LD_50_ values of 1.47 and 6.99 mg/adult, respectively [186]. In addition, the acaricidal ability of 4-methoxysalicylaldehyde (**294**) was greater than that of synthetic acaricides [187].

Zeng’s group discovered periplocoside X (**80**), which induced severe time-dependent cytotoxicity in the midgut epithelial cells of the red imported fire ant and inhibited amylase activity (*Solenopsis invicta*). Therefore, the ant insect midgut was deduced to be a target of periplocoside X [188]. It is noteworthy that, in addition to periplocoside X (**80**), there are some other periplocosides capable of impacting the digestive system of insects. Hu’s group has made vital contributions to the discovery of these periplocosides and has clarified the underlying mechanism. Periplocoside NW (**82**) [48,189,190] displayed potent stomach toxicity against some insect pests, including third-instar larvae of *P. xylostella*, *Mythimna separata* larva and *Pieris rapae*. The membrane system in insect midgut epithelial cells was the initial action site of periplocoside NW against insects. In 2012, periplocoside D (**50**) and F (**52**) were both detected by bioassay tests, and they showed stomach toxicity activities against third-instar larvae of *M. separata* with the LC_50_ values of 0.39 and 0.34 mg/mL, and against third-instar larvae of *P. xyllostella* with the LC_50_ values of 1.21 and 1.39 mg/mL, respectively [191]. The insecticidal activities against the third-instar larvae of *Pseudaletia separata* and *P. xylostella* of periplocoside P (**81**) were detected by an insecticidal activity bioassay.

To explore the insecticidal mechanisms of these compounds, some studies were implemented by Hu’s group. Periplocoside P caused toxicity toward *M. separate* though activating the weakly alkaline trypsin-like protease [192] and inhibiting the activity of cytochrome P450 in the midgut. Additionally, glutathione-S-transferase might be involved in insect detoxification [193]. Through their in-depth research exploring new pesticides and their action mechanisms against insects, Hu’s group found that the apical and basolateral membrane voltage (*V*_a_) rapidly decreased in the presence of periplocoside P in a time- and dose-dependent manner, similar to the effects of Cry1Ab. They speculated that periplocoside P adjusted the transport mechanisms at the apical membrane of the midgut epithelial cells by inhibiting the V-type H^+^ ATPase [194]. In addition, the V-type ATPase A subunit in the midgut epithelium might be regarded as the target binding site of periplocosides, which provided preliminary evidence for the mode of action of periplocosides [195]. Furthermore, aminopeptidases N and N3 of the midgut epithelium cells of *M. separata* larvae were extrapolated as potential targets of periplocoside E (**47**) by affinity chromatography [196].

### 4.6. Other Abilities

As early as the 1980s, glycosides K, H_1_ and H_2_ from *P. sepium* showed the potentiation of NGF-mediated nerve fiber outgrowth in organ cultures of chicken embryonic dorsal roots and sympathetic ganglia, and glycoside H_2_ (**97**) had the best effect [53]. Later, in 2012, the alcohol extract of *P. forrestii* led to a good reparation of nerve injury in *Pristionchus pacificus* caused by thermal stimulation in the concentration range of 2–16 mg/mL, while obvious toxicity was also detected when the concentration was increased [197]. Intriguingly, the sedative and potent analgesic effects of the methanolic extract of *P. aphylla* at the dose of 500 mg/kg were shown in a series of animal experiments [198].

*P. forrestii*, a folk medicine used to cure wounds, is also equipped with wound-healing activity. Zhang’s group [199] evaluated its ability through the proliferation of fibroblasts, migration and collagen production using L929 cells. The 65% ethanol eluate mainly composed of cardiac glycosides was the most effective fraction, suggesting that the cardiac glycosides may be the potential active ingredients in wound healing. In further research, periplocin (**1**) demonstrated its ability to significantly boost proliferation and migration and stimulate collagen production in fibroblast L929 cells. Its mechanism of promoting wound healing was intensely associated with the activation of the Src/ERK and PI3K/Akt pathways mediated by Na/K-ATPase [200]. Moreover, Abdel-Monem and co-workers [10] reported that ursolic acid played an important role in the protective effect against CCl_4_-induced injury on human hepatoma cell line (Huh7). Additionally, the antiacetylcholinesterase activities of triterpenes **199** and **198** with esterification of the alcohol function at C-3 were lower than that of lupeol (**197**). Ben Jannet’s group speculated that the triterpenic skeleton and the free secondary alcohol function at C-3 could be responsible for the inhibition ability [87]. In addition to the abovementioned abilities, both lipoxygenase inhibitory activity [84] and antimalarial activity [201] of this genus were detected but without further studies on the mechanisms of these actions.

## 5. Toxicology

Excessive or prolonged use of *P. sepium* leads to toxic effects, mainly consisting of cardiotoxicity and hepatorenal toxicity. Acute toxicity tests in mice and long-term toxicity tests in rats showed that the toxicity of ethanol extract from the root bark of *P. sepium* was greater than that of the water extract [202,203].

The water extract from the root bark of *P. sepium* was dried into powder, in which the content of periplocin was 21.91 mg/g. After the water extract was injected intraperitoneally into guinea pigs, their electrocardiograms showed obvious changes, and the incidence of abnormal electrocardiograms was proportional to the dose [204]. Sun’s group found that within seven days after oral administration, the water extract (0.78–15.0 g/kg) and alcohol extract (0.78–6.0 g/kg) from the root bark of *P. sepium* both caused obvious hepatotoxic damage in mice, which was represented by increased levels of alanine transaminase, aspartate transaminase, alkaline phosphatese and total bilirubin and a decreased content of albumin, as well as increases of the ratio of the liver to kidneys to different degrees [205]. The pathway of liver injury was inferred to be related to lipid peroxidation induced by oxidative stress [206]. Furthermore, periplocin (**1**) was revealed to have toxic effects at a certain dose. Deng and co-workers [207] detected that the maximum tolerable dose for the oral administration of periplocin in mice was 103 mg/kg. Periplocin was injected intraperitoneally into mice, resulting in an LD_50_ value of 15.20 mg/kg. In addition, periplocin at 0.39 mg/kg brought about electrocardiogram abnormalities in half of the tested guinea pigs. Noteworthy is that the content of periplocin had a tight correlation with the acute toxicity test results of the water extract from the root bark of *P. sepium*, while the content of 4-methoxysalicylaldehyde (**294**), a quality control index of Cortex Periplocae in the *Chinese Pharmacopoeia* (2015 version), did not directly affect the results of acute toxicity [208].

It was demonstrated that the oral bioavailability of periplocin (**1**) in rats was low [209]. Gao’s group [210] studied periplocin in the field of pharmacokinetics. After a single oral administration of periplocin at 50 mg/kg to rats, it was found that periplocin could not be detected at any time point, while the mean plasma profiles of its two metabolites periplocymarin (**3**) and periplogenin (**2**) were regular. Intriguingly, two metabolites participated in the pharmacokinetic process, and the main constituent in the blood was periplocymarin. In addition, periplocin could reach its peak concentration quickly after oral administration, whereas the two metabolites reached maximum concentrations more than 4.83 h after administration. It is worth noting that the tissue distributions of periplocin and two metabolites were found to include the heart, liver, spleen, lung and kidneys, but a small amount of chemical constituents were distributed in the brain [211].

To date, some toxicity studies on cardiac glycosides from the genus *Peripioca* have been carried out, but toxicity tests on other compounds are absent. To improve the safety of clinical therapy, comprehensive and in-depth investigations of the pharmacology and toxicity of plants of the genus *Peripioca* are necessary.

## 6. Conclusions

In this review, the botanical classification, chemical constituents, biological activities and toxicology studies of *Periploca* species were systematically summarized. There are differences in the classification of this genus at home and abroad, and some new species have not yet been included in flora. Currently, 314 diverse metabolites have been isolated from the genus *Periploca,* and are they are composed of steroids, carbohydrates, terpenoids, phenylpropanoids, flavonoids, quinones, aromatics, and so on. Steroids obtained from the genus *Periploca* account for the largest proportion. Moreover, these steroids, mainly composed of C_21_ pregnane and cardiotonic steroids, should be considered the characteristic compounds of this genus. Oligosaccharides, which have a high rate of new compounds (78.3%) featuring multifarious deoxysugar and non-deoxysugar, strictly conform to the regularity of the saccharide chains in the cardenolides and C_21_ pregnane glycosides of this genus. Hence, these oligosaccharides could also be regarded as representative compounds of this genus. Furthermore, *Periploca* species are rich in aromatic compounds and volatile oils, especially *P. sepium*. However, only 4-methoxy salicylaldehyde is assigned as the index component for the quality evaluation of *P. sepium* root bark in the *Chinese Pharmacopoeia* (2015 Version), which requires its content to be not less than 0.20% [3]. Other components characterized by a high content, favorable pharmacological activity or potential toxicity should perhaps also be used as legal quality evaluation indicators.

In regard to biological activities, the genus *Periploca* has received increasing attention over the last fifteen years. The relationships between various activities and their main related plant sources and active ingredients are concisely concluded. It is worth noting that periplocin is a research hotspot and has been demonstrated to possess various activities, such as cardiotonic, antitumor, immunosuppressive and wound-healing effects. However, periplocin has hepatotoxicity and cardiotoxicity at certain doses, suggesting that further pharmacological, toxicological and pharmacokinetics studies on this active compound are necessary. In addition, a series of periplosides with fascinating immunosuppressive and insecticidal properties should be another research focus. In view of periplosides’ available immunosuppressive effect in vitro, detailed animal experiments in vivo and the structure-activity relationships of the periplosides should also be studied to identify new immunosuppressants for the treatment of RA. More importantly, periplosides may be promising insecticidal alternatives, which is of great value to agriculture. Thus far, most experiments of biological activities of the genus *Periploca* have been conducted in vitro. Taking into account the important medical and agricultural value of this genus, intensive studies on the bioactivity-guided chemical isolation, pharmacological activities in vivo, and underlying mechanisms of the biological activities of this genus should be further performed.

## Figures and Tables

**Table 1:** Thirteen studied species of the genus *Periploca*.

Name	Ref.
*P. sepium* Bunge	[2,7]
*P. forrestii* Schlechter	[2]
*P. graeca* L	[7]
*P. calophylla* (Wight) Falconer	[2,7]
*P. aphylla* Decne	[7]
*P. nigrescens* Afz.	[7]
*P. linearifolia,* Quart.-Dill. and A. Rich	[7]
*P. laevigata* Ait	[7]
*P. hydaspidis* Falc	[7]
*P. angustifolia* Labill	[7]
*P. somaliensis* Browicz	[10]
*P. omeiensis* Z. Y. Zhu	[8]
*P. chrysantha* D. S. Yao, X. C. Chen et J. W. Ren	[9]

**Table 2:** Steroids from the genus *Periploca*.

No.	Compounds	Sources	Parts of Plants	Ref.
***Cardenolide-type steroids***				
1	periplocin	*P. graeca* *P. omeiensis* *P. calophylla* *P. forrestii* *P. sepium*	bark whole plant stems whole plant cortex	[11] [8] [12] [13] [14]
2	periplogenin	*P. graeca* *P. calophylla* *P. forrestii* *P. sepium*	bark, stems stems whole plant cortex	[11,15] [12] [13] [16]
3	periplocymarin	*P. graeca* *P. sepium* *P. forrestii*	stalks and bark cortex whole plant	[15,17] [14] [13]
4	biondianoside A	*P. graeca*	stems	[15]
5	periplogenin 3-*O*-*β*-d-glucopyranoside	*P. graeca*	stems	[15]
6	periplogenin 3-[*O*-*β*-glucopyranosyl-(1→4)-*β*-sarmentopyranoside]	*P. sepium*	root bark	[18]
7	periplogenin 3-*O*-*β*-d-glucopyranosyl-(1→4)-*O*-*β*-D-digitoxopyranoside	*P. forrestii*	stems	[19,20]
8	periplogenin 3-*O*-*β*-d-glucopyranosyl-*β*-digitaloside	*P. forrestii*	stems	[20]
9	periplogenin 3-*O*-*β*-d-digitoxopyranoside	*P. forrestii*	stems	[19]
10	7*β*-hydroxy-periplogenin	*P. forrestii*	stems	[19]
11	7-hydroxyl-periplogenin 3-*O*-*β*-d-digitoxopyranoside	*P. forrestii*	stems	[20]
12	7-hydroxyl-periplogenin 3-*O*-*β*-d-cymaropyranoside	*P. forrestii*	stems	[20]
13	7-hydroxyl-periplogenin 3-*O*-*β*-d-glucopyranosyl-*β*-d-digitoxopyranoside	*P. forrestii*	stems	[20]
14	8*β*-hydroxy-periplogenin	*P. forrestii*	rhizome, stems	[19,21]
15	8-hydroxyl-periplogenin 3-*O*-*β*-d-digitoxopyranoside	*P. forrestii*	stems	[20]
16	periforoside G	*P. forrestii*	stems	[19]
17	periforoside H	*P. forrestii*	stems	[19]
18	7,8-dihydroxyl-periplogenin 3-*O*-*β*-d-cymaropyranoside	*P. forrestii*	stems	[20]
19	periforgenin C	*P. forrestii*	stems	[19]
20	periforoside F	*P. forrestii*	stems	[19]
21	7,8-epoxy-periplogenin 3-*O*-*β*-d-glucopyranosyl-*β*-d-cymaropyranoside	*P. forrestii*	stems	[20]
22	periploforgeside A	*P. forrestii*	unknown	[22]
23	periploforgeside B	*P. forrestii*	unknown	[22]
24	echubioside	*P. graeca*	stems	[15]
25	xysmalogenin	*P. sepium*	root bark	[23]
26	3*β*,5*β*-dihydroxy-14-en-card-20(22)-enolide	*P. forrestii*	stems	[19]
27	periforoside E	*P. forrestii*	stems	[19]
28	strophanthidin	*P. nigrescens*	branches	[24,25]
29	strophanthidol	*P. nigrescens*	branches	[24]
30	strophanthidin-*β*-d-glucoside	*P. nigrescens*	branches	[26]
31	strophadogenin	*P. nigrescens*	branches	[25]
32	convallatoxin	*P. nigrescens*	branches	[25]
33	16*β*-acetoxystrophanthidin	*P. nigrescens*	branches	[27]
34	3-*O*-rhamnosyl-16*β*-acetoxystrophanthidin	*P. nigrescens*	branches	[27]
35	16-dehydrostrophanthidin	*P. nigrescens*	branches	[27]
36	16-dehydrostrophanthidol	*P. nigrescens*	branches	[27]
37	3-*O*-digitoxosyl-16-dehydrostrophanthidin	*P. nigrescens*	branches	[27]
38	alloperiplogenin 3-*O*-*β*-d-glucopyranosyl-(1→4)-*β*-d-cymaropyranoside	*P. graeca*	stems	[15]
39	alloperiplogenin	*P. graeca*	stems	[15]
40	biondianoside B	*P. graeca*	stems	[15]
41	periforgenin A	*P. forrestii*	rhizome, stems	[19]
42	periforoside I	*P. forrestii*	rhizome, stems	[19,28]
43	periforgenin A 3-*O*-*β*-cymaropyranoside	*P. forrestii*	roots	[29]
44	periforgenin A-3-*O*-*β*-digitoxopyranoside	*P. forrestii*	stems	[30]
45	periforoside D	*P. forrestii*	stems	[19]
46	periforgenin 3-*O*-*β*-d-glucopyranosyl-*β*-d-glucopyranosyl-*β*-d-cymaropyranoside	*P. forrestii*	stems	[20]
***C_21_ pregnane-type steroids***				
47	periploside A (periplocoside E)	*P. sepium* *P. calophylla*	root bark stems	[31,32,33,34,35] [12]
48	periploside B	*P. sepium*	root bark	[32]
49	periploside C (periplocoside A)	*P. sepium*	root bark	[32,33,34,35,36]
50	periploside D (periplocoside D)	*P. sepium*	root bark	[31,33,34,35]
51	periploside E (periperoxide A)	*P. sepium*	root bark	[33,34,35]
52	periploside F (periplocoside F)	*P. sepium*	root bark	[33,34,35,37]
53	periploside G (periperoxide B)	*P. forrestii*	root bark	[33,34,35]
54	periploside H (periperoxide E)	*P. forrestii*	root bark	[33,34,35]
55	periploside I (periperoxide C)	*P. forrestii*	root bark	[33,34,35]
56	periploside J (periplocoside J)	*P. sepium*	root bark	[34,35,37]
57	periploside K (periplocoside K)	*P. sepium*	root bark	[34,35,37]
58	periploside L (periperoxide D)	*P. forrestii*	root bark	[33,34,35]
59	periploside M (periplocoside B)	*P. sepium*	root bark	[34,35,36]
60	periploside N (periplocoside C)	*P. sepium*	root bark	[34,35,36]
61	periploside O	*P. sepium*	root bark	[38]
62	periploside P	*P. sepium*	root bark	[38]
63	periploside Q	*P. sepium*	root bark	[38]
64	periploside R	*P. sepium*	root bark	[38]
65	periploside S	*P. sepium*	root bark	[38]
66	periploside T	*P. sepium*	root bark	[38]
67	periploside U	*P. sepium*	root bark	[38]
68	periploside V	*P. sepium*	root bark	[38]
69	3-*O*-formyl-periploside A	*P. sepium*	root bark	[38]
70	periploside W	*P. chrysantha*	root bark	[39]
71	periploside X	*P. chrysantha*	root bark	[39]
72	periploside Y	*P. chrysantha*	root bark	[39]
73	3-*O*-formyl-periploside F	*P. chrysantha*	root bark	[39]
74	Δ^5^-pregnene-3*β*,17*α*,20(*S*)-triol	*P. sepium*	root bark	[31,40]
75	periplocogenin	*P. sepium*	root bark	[41]
76	periplocoside L	*P. sepium* *P. forrestii*	root bark rhizome	[31] [42]
77	periplocoside M periploside B	*P. sepium* *P. forrestii* *P. calophylla* *P. sepium*	root bark stems stems root bark	[31,43] [30] [12] [44]
78	periplocoside N	*P. sepium* *P. forrestii*	root bark root bark	[31] [45]
79	periplocoside O	*P. sepium*	root bark	[37]
80	periplocoside X	*P. sepium*	roots	[46]
81	periplocoside P	*P. sepium*	root bark	[47]
82	periplocoside NW	*P. sepium*	root bark	[48]
83	(3*β*,20*S*)-pregn-5-ene-3,17,20-triol 20-[*O*-*β*-glucopyranosyl-(1→6)-*O*-glucopyranosyl-(1→4)-*β*-canaropyranoside]	*P. sepium*	root bark	[18]
84	periseoside A	*P. sepium*	root bark	[40]
85	periseoside B	*P. sepium*	root bark	[40]
86	perisepiumoside I	*P. sepium*	root bark	[43]
87	Δ^5^-pregnene-3*β*,20(*S*)-diol	*P. sepium*	root bark	[44,49]
88	Δ^5^-pregnene-3*β*,20(*S*)-diol 3-*O*-[*β*-d-digitalopyranosyl (1→4)*β*-d-cymaropyranoside] 20-*O*-[*β*-d-glucopyranosyl (l→6)-*β*-d-glucopyranosyl (1→2)-*β*-d-digitalopyranoside]	*P. sepium* *P. graeca*	root bark small branches	[49] [50]
89	Δ^5^-pregnene-3*β*,20(*S*)-diol 3-*O*-[2-*O*-acetyl-*β*-d-digitalopyranosyl (1→4)-*β*-d-cymaropyranoside] 20-*O*-[*β*-d-glucopyranosyl (l→6)-*β*-d-glucopyranosyl (l→2)-*β*-d-digitalopyranoside]	*P. sepium*	root bark	[49]
90	plocoside A	*P. sepium*	root bark	[49,51]
91	biondianoside C	*P. sepium*	root bark	[52]
92	biondianoside D	*P. sepium*	root bark	[52]
93	periseoside C	*P. sepium*	root bark	[40]
94	periseoside D	*P. sepium*	root bark	[40]
95	5-pregnen-3*β*,20*β*-diol glucoside	*P. sepium*	root bark	[43]
96	Δ^5^-pregnene-3*β*,20(*R*)-diol 3-*O*-monoacetate	*P. sepium*	root bark	[31]
97	glycoside H_2_	*P. sepium* *P. graeca*	cortex, root bark small branches	[40,49,53] [50]
98	Δ^5^-pregnene-3*β*,16*α*,20(*S*)-triol	*P. sepium*	root bark	[16,49]
99	Δ^5^-pregnene-3*β*,16*α*,20(*S*)-triol 20-*O*-*β*-d-glucopyranosyl (1→6)-*β*-d-glucopyranosyl (l→2)-*β*-d-digitalopyranoside	*P. sepium*	root bark	[49,52]
100	plocoside B	*P. sepium*	root bark	[40,51]
101	periseoside E	*P. sepium*	root bark	[40]
102	16*α*-[(6-*O*-sulfo-*β*-d-glucopyranosyl)oxy] pregn-5-en-20-ol-3*β*-yl *O*-(2-*O*-acetyl-*β*-d-digitalopyranosyl)-(1→4)-*β*-d-cymaropyranoside	*P. graeca*	small branches	[50]
103	16*α*-[(6-*O*-sulfo-*β*-d-glucopyranosyl) oxy] pregn-5-en-20-ol-3*β*-yl *O*-*β*-d-oleandropyranosyl-(1→4)-*β*-d-oleandropyranoside	*P. graeca*	small branches	[50]
104	16*α*-[(6-*O*-sulfo-*β*-d-glucopyranosyl)oxy] pregn-5-en-20-ol-3*β*-yl *O*-*β*-d-oleandropyranoside	*P. graeca*	small branches	[50]
105	16*α*-[(6-*O*-sulfo-*β*-D-glucopyranosyl)oxy] pregn-5-ene-3*β*,20-diol	*P. graeca*	small branches	[50]
106	20-*O*-[(*β*-d-glucopyranosyl-(1→6)-*β*-d-glucopyranosyl-(1→2)-*β*-d-digitalopyranosyl)oxy] pregn-5-en-16*β*-ol-3*β*-yl *O*-*β*-d-digitalopyranosyl-(1→4)-*β*-d-cymaropyranoside	*P. graeca*	small branches	[50]
107	3*β*,16*β*,20-trihydroxy-pregn-5-en-3-*O*-2,4-diacetyl-*β*-digitalopyranosyl-(1→4)-*O*-*β*-cymaropyranosyl-20-*O*-*β*-glucopyranosyl-(1→6)-*O*-*β*-glucopyranosyl-(1→2)-*O*-*β*-digitalopyranoside	*P. forrestii*	roots	[29]
108	Δ^5^-pregnene-3*β*,16*β*,20(*R*)-triol	*P. sepium*	root bark	[49]
109	Δ^5^-pregnene-3*β*,16*β*,20(*R*)-triol 3-*O*-[2-*O*-acetyl-*β*-d-digitalopyranosyl (1→4)-*β*-d-cymaropyranoside] 20-*O*-[*β*-d-glucopyranosyl (l→6)-*β*-d-glucopyranosyl (l→2)-*β*-d-digitalopyranoside]	*P. sepium* *P. graeca*	root bark small branches	[49] [50]
110	Δ^5^-pregnene-3*β*,16*β*,20(*R*)-triol 20-*O*-*β*-d-glucopyranosyl (l→6)-*β*-d-glucopyranosyl (l→2)-*β*-d-digitalopyranoside	*P. sepium*	root bark	[49]
111	calocin	*P. calophylla*	twigs	[54]
112	plocin	*P. calophylla*	twigs	[55]
113	plocigenin	*P. calophylla*	twigs	[55]
114	locin	*P. calophylla*	twigs	[56]
115	calocinin	*P. calophylla*	twigs	[57]
116	calogenin 3-*O*-*β*-d-digitalopyranoside-20-*O*-*β*-d-canaropyranoside	*P. graeca*	small branches	[50]
117	(3*β*,14*β*)-3,14-dihydroxy-21-methoxypregn-5-en-20-one	*P. sepium*	root bark	[44]
118	perisepiumosides E	*P. sepium*	root bark	[58]
119	(3*β*,14*β*,17*α*)-3,14,17-trihydroxy-21-methoxypregn-5-en-20-one 3-[*O*-*β*-oleandropyranosyl-(1→4)-*O*-*β*-cymaropyranosyl-(1→4)-*β*-cymaropyranoside]	*P. sepium*	root bark	[18]
120	(3*β*,14*β*,17*α*)-3,14,17-trihydroxy-21-methoxypregn-5-en-20-one	*P. sepium*	root bark	[18]
121	perisepiumoside G	*P. sepium*	root bark	[43]
122	perisepiumoside H	*P. sepium*	root bark	[43]
123	calogenin	*P. calophylla*	unknown	[59]
124	plocinine	*P. calophylla*	twigs	[60]
125	21-*O*-methyl-5-pregnene-3*β*,14*β*,17*β*,20,21-pentaol	*P. sepium*	root bark	[23]
126	perisepiumosides A	*P. sepium*	root bark	[58]
127	perisepiumosides B	*P. sepium*	root bark	[58]
128	perisepiumoside F	*P. sepium*	root bark	[43]
129	(3*β*,14*β*,17*β*)-3,14,17-trihydroxy-21-methoxypregn-5-en-20-one	*P. sepium*	root bark	[23]
130	perisepiumosides C	*P. sepium*	root bark	[58]
131	perisepiumosides D	*P. sepium*	root bark	[58]
132	21-*O*-methyl-Δ^5^-pregnene-3*β*,14*β*,17*β*,21-tetraol-20-one-3-*O*-*β*-d-oleandropyranosyl(1→4)-*β*-d-cymaropyranosyl-(1→4)-*β*-d-cymaropyranosyl (periplocoside P)	*P. sepium*	root bark	[61]
133	21-*O*-methyl-5,14-pregndiene-3*β*,17*β*,20,21-tetraol	*P. sepium*	root bark	[23]
134	Δ^5,16^-pregnadiene-3*β*,20*α*-diol diacetate	*P. sepium*	root bark	[62]
135	neridienone A	*P. sepium*	root bark	[41]
136	5*α-*pregn-6-ene-3*β*,17*α*,20(*S*)-triol-20-*O*-*β*-d-digtoxopyranoside	*P. forrestii*	stems	[63]
137	teikagenin-3-*O*-*β*-d-dignitalosyl-20-*O*-*β*-d-canaroside	*P. forrestii*	stems	[63]
138	3-*O*-*β*-d-digitalosyl-3*β*,17*α*,20*α*-tihydroxy-5*α*-pregn-6-ene	*P. forrestii*	stems	[63]
***Phytosterol-type steroids***				
139	*β*-sitosterol	*P. omeiensis* *P. calophylla* *P. laevigata* *P. forrestii* *P. aphylla* *P. linearifolia*	whole plant root bark roots stems whole plant stem bark	[8] [64] [65] [66] [67] [68]
140	*β*-daucosterol	*P. omeiensis* *P. calophylla* *P. forrestii* *P. sepium* *P. laevigata * *P. aphylla*	whole plant root bark root bark root bark fruit bark above ground part	[8] [64] [21] [62] [69] [70]
141	(6′-*O*-palmitoyl)-sitosterol-3-*O-β*-d-glucoside	*P. calophylla*	root bark	[71]
142	stigmasterol	*P. aphylla*	unknown	[72]

**Table 3:** Carbohydrates from the genus *Periploca*.

No.	Compounds	Sources	Parts of Plants	Ref.
143	cymarose	*P. calophylla*	twigs	[55]
144	*β*-d-glucopyranose	*P. laevigata*	roots	[65]
145	*α*-d-glucopyranose	*P. laevigata*	roots	[65]
146	sucrose	*P. sepium*	stems	[82]
147	4-*O*-(2-*O*-acetyl-*β*-d-digitalopyranosyl)-d-cymaropyranose	*P. sepium*	cortex, root bark	[49,83]
148	methyl 4-*O*-(2-*O*-acetyl-*β*-d-digitalopyranosyl)-*β*-d-cymaropyranoside	*P. sepium*	cortex	[83]
149	methyl *β*-d-digitalopyranosyl(1→4)-*β*-d-cymaropyranoside	*P. sepium*	root bark	[49]
150	oligosaccharide C_1_	*P. sepium*	cortex	[78]
151	oligosaccharide D_2_	*P. sepium*	cortex, root bark	[33,78]
152	oligosaccharide F_1_	*P. sepium*	cortex	[78]
153	oligosaccharide F_2_	*P. sepium*	cortex, root bark	[33,78]
154	perisaccharide A	*P. sepium*	root bark	[33]
155	perisaccharide B	*P. sepium* *P. calophylla*	root bark stems	[33,43] [81]
156	perisaccharide C	*P. sepium*	root bark	[33]
157	perifosaccharide A	*P. forrestii*	roots	[29]
158	perifosaccharide B	*P. forrestii*	roots	[29]
159	perifosaccharide C	*P. forrestii*	roots	[29]
160	perifosaccharide D	*P. forrestii*	roots	[29]
161	perisesaccharide A	*P. sepium*	root bark	[79]
162	perisesaccharide B	*P. sepium*	root bark	[43,79]
163	perisesaccharide C	*P. sepium*	root bark	[43,79]
164	perisesaccharide D	*P. sepium*	root bark	[79]
165	perisesaccharide E	*P. sepium*	root bark	[79]
166	perisesaccharide F	*P. sepium*	root bark	[80]
167	4-*O*-acetyl-*β*-cymaropyranosyl(1→4)-*O*-*β*-d-cymaropyranosyl(1→4)-*O*-*β*-d-canaropyranosyl(1→4)-*O*-*β*-d-cymaropyranosy(l→4)-*O*-oleandronic acid-*δ*-lactone	*P. calophylla*	stems	[81]
168	2-*O*-acetyl-*β*-D-digitalopyranosyl-(1→4)-*β*-d-cymaropyranosyl- (1→4)-*β*-d-cymaropyranosyl-(1→4)-*β*-d-cymaropyranosyl- (1→4)-*β*-d-oleandronic-*δ*-lactone	*P. sepium*	root bark	[43]

**Table 4:** Terpenoids from the genus *Periploca*.

No.	Compounds	Sources	Parts of Plants	Ref.
169	loliolide	*P. forrestii*	whole plant	[73]
170	periplocadiol	*P. laevigata*	roots	[65]
171	2*α*,6*α*-dihydroxy-5-[(*E*)-3′-hydroxy-3′-methyl-1′-butenyl]-6-methyl-4-cyclohexen-3-one	*P. aphylla*	whole plant	[84]
172	oleanolic acid	*P. forrestii* *P. sepium* *P. laevigata* *P. aphylla*	unknown leaves roots above ground part	[85] [86] [65] [70]
173	hederagenin	*P. omeiensis* *P. forrestii*	whole plant whole plant	[8] [13]
174	2*α*,3*β*,5,24-tetrahydroxy-olean-12-en-28-oic acid	*P. forrestii*	stems	[66]
175	masilinic acid	*P. laevigata* *P. aphylla*	fruit bark above ground part	[69] [70]
176	arjunolic acid	*P. laevigata*	latex	[87]
177	12*α*-hydroxy-*δ*-lactone of oleanolic acid	*P. laevigata*	latex	[87]
178	11*α*,12*α*-Epoxy-3*β*-hydroxy-olean-13*β*,28-olide	*P. somaliensis*	fruits	[10]
179	3-*O*-acety loleanolic acid	*P. forrestii*	stems	[88]
180	3*β*-hydroxy-11,13(18)-diene-olean-28-oicacid	*P. forrestii*	unknown	[85]
181	*β*-amyrin	*P. calophylla* *P. laevigata* *P. forrestii* *P. sepium* *P. linearifolia*	twigs roots unknown stem bark stem bark	[89] [65] [90] [91] [68]
182	*β*-amyrin acetate	*P. forrestii* *P. sepium*	unknown root bark, stem bark	[90] [92,93]
183	P1	*P. calophylla*	twigs	[89]
184	P2	*P. calophylla*	twigs	[89]
185	P3	*P. calophylla*	twigs	[89]
186	ursolic acid	*P. omeiensis* *P. calophylla* *P. forrestii* *P. somaliensis* *P. aphylla* *P. nigrescens*	whole plant rhizome rhizome fruits unknown leaves	[8] [64] [21] [10] [72] [94]
187	2*α*,3*β*-dihydroxy ursolic acid	*P. forrestii* *P. calophylla* *P. aphylla*	stems stems unknown	[88] [64] [72]
188	asiatic acid	*P. laevigata* *P. calophylla* *P. forrestii* *P. aphylla*	roots stems stems unknown	[65] [12] [30] [72]
189	12-ursen-3*β*-acetyl-11-one	*P. forrestii*	unknown	[85]
190	jacoumaric acid	*P. forrestii*	stems	[88]
191	14-ursen-3-ol-1-one	*P. forrestii*	stems	[88]
192	taraxasterol	*P. forrestii*	stems	[88]
193	*α*-amyrin	*P. forrestii* *P. sepium* *P. laevigata*	unknown root bark roots	[90] [92] [65]
194	27-hydroxy-*α*-amyrin	*P. omeiensis* *P. forrestii*	whole plant unknown	[8] [90]
195	*α*-amyrin acetate	*P. calophylla* *P. forrestii* *P. sepium*	twigs unknown root bark	[89] [90] [95]
196	3*β*-hydroxy-urs-11-en-13*β*,28-olide	*P. somaliensis*	fruits	[10]
197	lupeol	*P. laevigata* *P. aphylla*	roots, latex above ground part	[65,87] [70]
198	lupeol acetate	*P. calophylla* *P. laevigata* *P. sepium* *P. aphylla*	rhizome latex cortex above ground part	[64] [87] [95] [70]
199	lupeol arachidate	*P. laevigata*	latex	[87]
200	procrim a	*P. laevigata*	latex	[87]
201	procrim b	*P. laevigata* *P. linearifolia*	latex stem bark	[87] [68]
202	laevigatin I	*P. laevigata*	latex	[87]
203	laevigatin II	*P. laevigata*	latex	[87]
204	lupeol-20(29)-en-3-nonadecanoate	*P. forrestii*	roots	[96]
205	3*β*,6*α*-dihydroxylup-20(29)-ene	*P. aphylla*	stems	[67,72]
206	6*α*-hydroxylup-20(29)-en-3*β*-octadecanoate	*P. aphylla*	stems	[67,72]
207	betuline	*P. aphylla*	unknown	[72]
208	(24*R*)-9,19-cycloart-25-ene-3*β*,24-diol	*P. sepium*	root bark	[92]
209	(24*S*)-9,19-cycloart-25-ene-3*β*,24-diol	*P. sepium*	root bark	[92]
210	cycloeucalenol	*P. sepium*	root bark	[92]

**Table 5:** Phenylpropanoids from the genus *Periploca*.

No.	Compounds	Sources	Parts of Plants	Ref.
211	sinapic acid	*P. calophylla*	rhizome	[71]
212	sinapate glucose-1-ester	*P. calophylla*	rhizome	[99,100]
213	*E*-*p*-hydroxy-cinnamic acid	*P. forrestii*	stems	[88]
214	caffeic acid	*P. forrestii*	stems	[88]
215	ethyl caffeate	*P. forrestii* *P. sepium*	stems root bark	[101] [98]
216	(*E*)-1-(2,4-dihydroxy phenyl)-ethyl acrylate	*P. forrestii*	stems	[101]
217	*trans*-3,4-methylenedioxy cinnamyl alcohol	*P. forrestii*	whole plant	[73]
218	6′-*O*-(*E*)-feruloyl sucrose	*P. forrestii*	whole plant	[73]
219	3-*O*-caffeoylquinic acid	*P. forrestii* *P. sepium*	roots and stems roots	[74] [102]
220	4-*O*-caffeoylquinic acid	*P. forrestii*	roots and stems	[74]
221	5-*O*-caffeoylquinic acid	*P. forrestii*	roots and stems	[74]
222	3*-O*-caffeoylquinic acid methyl ester	*P. forrestii*	roots and stems	[74]
223	4-*O*-caffeoylquinic acid methyl ester	*P. forrestii*	roots and stems	[74]
224	5-*O*-caffeoylquinic acid methyl ester	*P. forrestii*	roots and stems	[74]
225	1,3-di-*O*-caffeoylquinic acid	*P. forrestii*	roots and stems	[74]
226	3,4-di-*O*-caffeoylquinic acid	*P. forrestii*	roots and stems	[74]
227	3,5-di-*O*-caffeoylquinic acid	*P. forrestii*	roots and stems	[74]
228	4,5-di-*O*-caffeoylquinic acid	*P. forrestii*	roots and stems	[74]
229	scopoletin	*P. forrestii* *P. sepium* *P. nigrescens*	whole plant cortex and seedlings bark, leaves, seeds	[73,85] [103] [97]
230	cleomiscosin A	*P. forrestii* *P. calophylla*	root bark, stems rhizome	[45,66] [71]
231	cleomiscosin B	*P. forrestii*	stems	[66]
232	(+)-lyoniresinol	*P. aphylla*	whole plant	[84]
233	(+)-lyoniresinol-3*α*-*O*-*β*-d-glucopyranoside	*P. forrestii*	stems	[63]
234	(-)-lyoniresinol-3α-*O*-*β*-d-glucopyranoside	*P. forrestii*	stems	[63]
235	tortoside B	*P. sepium*	root bark	[98,104]
236	(-)-gentioluteol-9-*O*-*β*-d-glucoside	*P. forrestii*	stems	[63]
237	(-)-berchemol 9-*O*-*β*-d-apiofuranosyl-(1→6)-*O*-*β*-d-glucopyranoside	*P. forrestii*	stems	[63]
238	(7*S*,8*R*)-dihydrodehydrodiconiferyl alcohol 9-*O*-*β*-d-apiofuranosyl-(1→6)- *O*-*β*-d-glucopyranoside	*P. forrestii*	stems	[63]
239	7*S*,7′*S*,8*R*,8′*R*-lcariol A2-9-*O*-*β*-d-glucopyranoside	*P. forrestii*	stems	[63]
240	(+)-1-hydroxypinoresinol	*P. omeiensis* *P. forrestii*	whole plant whole plant	[8] [13]
241	syringaresinol	*P. forrestii*	whole plant	[13,73]
242	(+)-syringaresinol-4′-*O*-*β*-d-monoglucoside	*P. forrestii* *P. calophylla*	whole plant rhizome	[73] [99,105]

**Table 6:** Flavonoids from the genus *Periploca*.

No.	Compounds	Sources	Parts of Plants	Ref.
243	kaempferol	*P. forrestii*	unknown	[85]
244	kaempferol-3-*O*-*α*-d-arabinopyranoside	*P. calophylla*	rhizome	[99]
245	kaempferol-3-*O*-*β*-d-glucopyranoside	*P. calophylla* *P. laevigata* *P. graeca*	rhizome aerial parts bark, leaves, and seeds	[99] [106] [97]
246	kaempferol-3-*O*-*β*-d-galactopyranoside	*P. forrestii*	stems	[107]
247	kaempferol-3-*O*-*β*-l-arabinopyranoside	*P. forrestii* *P. laevigata*	stems aerial parts	[107] [106]
248	rutin	*P. laevigata* *P. aphylla * *P. graeca*	aerial parts aerial parts bark, leaves, and seeds	[106] [108] [97]
249	quercetin-3-*O*-*β*-d-glucopyranoside	*P. forrestii* *P. sepium* *P. laevigata* *P. aphylla * *P. graeca*	stems leaves aerial parts aerial parts bark, leaves, and seeds	[107] [86] [106] [108] [97]
250	quercetin	*P. forrestii* *P. sepium*	unknown leaves	[85] [86]
251	quercetin-3-*O*-*β*-l-arabinopyranoside	*P. forrestii*	stems	[107]
252	6′-Methyl ester of quercetin-3-*O*-*β*-d-glucuronide	*P. sepium*	leaves	[86]
253	quercetin-3-*O*-*α*-l-rhamnopyranoside	*P. aphylla*	aerial parts	[108]
254	quercetin-3-*O*-*α*-l-arabinopyranoside	*P. forrestii*	unknown	[109]
255	baohuoside I	*P. sepium*	cortex	[110]
256	quercetin-7-*O*-*β*-d-glucopyranoside	*P. forrestii*	unknown	[109]
257	apigenin	*P. nigrescens*	leaves	[94]
258	isorhoifolin	*P. nigrescens*	leaves	[94]
259	wogonin	*P. forrestii*	rhizome	[42]
260	negletein	*P. forrestii*	rhizome	[42]
261	liquiritigenin	*P. forrestii*	root bark	[45]
262	3′,4′,5,7-tetrahydroxy-flavanone-2(*S*)-3′-*O*-*β*-d-glucopyranoside	*P. calophylla*	rhizome	[99]
263	isoliquiritigenin	*P. forrestii*	root bark	[45]
264	daidzein	*P. forrestii*	root bark	[45]
265	formononetin	*P. forrestii*	root bark	[45]
266	(−)-epicatechin	*P. forrestii*	stems	[63]
267	cinchonain Ia	*P. forrestii*	stems	[63]
268	cinchonain Ib	*P. forrestii*	stems	[63]
269	proanthocyanidin A_2_	*P. forrestii*	stems	[63,88]
270	aesculitannin B	*P. forrestii*	stems	[63]
271	catechin-(3′→*O*→3′’’)-afzelechin	*P. aphylla*	whole plant	[84]
272	epicatechin-(3′→*O*→7′’)-epiafzelechi	*P. aphylla*	whole plant	[84]
273	(−)-maackiain	*P. forrestii*	root bark	[45]

**Table 7:** Quinones from the genus *Periploca*.

No.	Compounds	Sources	Parts of Plants	Ref.
274	physcion	*P. calophylla* *P. forrestii*	rhizome rhizome, root bark	[64] [45,112]
275	physcion-8-*O*-*β*-d-glucopyranoside	*P. forrestii*	stems	[107]
276	emodin	*P. forrestii*	stems	[90]
277	emodin-8-*O*-*β*-d-glucopyranoside	*P. forrestii*	stems	[107]
278	emodin-8-methyl ether	*P. forrestii*	stems	[66]
279	chrysophanol	*P. forrestii*	root bark	[45]
280	1,3,6-trihydroxy-2,5-dimethoxyxanthone	*P. aphylla*	whole plant	[84]
281	tanshinone IIA	*P. forrestii*	unknown	[111]

**Table 8:** Aromatics from the genus *Periploca*.

No.	Compounds	Sources	Parts of Plants	Ref.
282	vanillic acid	*P. calophylla* *P. forrestii*	rhizome unknown	[64] [66]
283	protocatechuic acid	*P. forrestii*	root bark	[45]
284	salicylic acid	*P. calophylla* *P. forrestii*	rhizome roots and stems	[71] [74]
285	4-methoxysalicylic acid	*P. sepium*	root bark	[92]
286	syringic acid	*P. forrestii*	stems, roots and stems	[74,90]
287	*p*-hydroxybenzoic acid	*P. forrestii*	roots and stems	[74]
288	2,4-dihydroxybenzoic acid methyl ester	*P. forrestii*	whole plant	[13]
289	2-ethylhexyl benzoate	*P. aphylla*	aerial parts	[108]
290	*o*-phthalic acid bis(2-ethylnonyl) ester	*P. aphylla*	whole plant	[84]
291	erigeside C	*P. calophylla*	rhizome	[99]
292	4′-hydroxy-3′-methoxy-phenol-*β*-d-[6-*O*-9-(4′’-hydroxy-3′’,5′’-dimethoxy benzoate)] glucopyranoside	*P. forrestii*	stems	[63]
293	4-hydroxy-2-methoxyphenyl 6′-*O*-syringoyl-*β*-d-glucopyranoside	*P. forrestii*	stems	[63]
294	4-methoxy salicylaldehyde	*P. graeca* *P. sepium*	bark root bark	[113] [16,114]
295	4-methoxybenzaldehyde-2-*O*-[*β*-d-xylopyranosyl-(1→6)-*β*-d-glucopyranoside]	*P. sepium*	root bark	[115]
296	4-hydroxy-3,5-dimethoxy benzaldehyde	*P. calophylla*	rhizome	[64]
297	4-hydroxy-3-methoxyl benzaldehyde	*P. calophylla* *P. sepium* *P. forrestii*	rhizome root bark whole plant	[71] [92] [73]
298	4-methoxy salicylaldehyde lactoside	*P. sepium*	root bark	[93]
299	isovanillin	*P. sepium* *P. forrestii*	root bark rhizome	[92] [42]
300	protocatechuic aldehyde	*P. forrestii*	roots and stems	[74]
301	2-hydroxy-5-(2-hydroxy-4-methoxybenzyl)-4-methoxybenzaldehyde	*P. sepium*	root bark	[44]
302	3-propyl anisole	*P. forrestii*	rhizome	[116]
303	2,6-dimethoxy-4-hydroxyphenol-1-*O*-*β*-d-glucoside	*P. calophylla*	rhizome	[116]
304	periplocain A	*P. aphylla*	aerial parts	[108]

**Table 9:** Other compounds from the genus *Periploca*.

No.	Compounds	Sources	Parts of Plants	Ref.
305	1-*O*-*β*-d-glucopyranosyl-(2*S*,3*S*,4*R*,10*E*)-2-[(2*R*)-2-hydroxytetracosanoylamino]-10-octadecene-3,4-diol	*P. forrestii*	whole plant	[116]
306	(2*S*,3*S*,4*R*,10*E*)-2-[(2*R*)-2-hydroxytetracosanoylamino]-10-octadecene-1,3,4-triol	*P. forrestii*	whole plant	[116]
307	N-(4-ethoxyphenyl)-acetamide	*P. forrestii*	rhizome	[112]
308	malonic acid	*P. forrestii*	whole plant	[13]
309	palmitic acid	*P. forrestii*	unknown	[85]
310	*n*-heptadecane	*P. forrestii*	unknown	[85]
311	1-triacontanol	*P. calophylla*	rhizome	[71]
312	methyl (9*S*,12*S*,13*S*)-9,12,13-trihydroxy-10*E*-octadecenoate	*P. forrestii*	stems	[63]
313	methyl (9*S*,12*R*,13*S*)-9,12,13-trihydroxy-10*E*-octadecenoate	*P. forrestii*	stems	[63]
314	obaculactone	*P. sepium*	root bark	[80]

**Table 10:** Biological activities of the genus *Periploca*, primarily interrelated species and active components.

Biological Activities	Interrelated Species ^1^	Primarily Active Components
cardiotonic effect	b, c, d	periplocin
anti-inflammatory and immunosuppressive effects	a	periplosides; periplocin; several triterpenoids
antitumor ability	a, b, d, e	periplocin; periplocymarin; several pregnane glycosides; several oligosaccharides; baohuoside I; lupeol acetate
antimicrobial and antioxidant abilities	a, b, e, f	4-methoxy salicylaldehyde; several lignins; several flavanes; several polysaccharides
insecticidal activity	a	4-methoxysalicylaldehyde; periplosides
other abilities	a, b	several pregnane glycosides; several cardenolides; several triterpenes

^1^ a: *P. sepium*; b: *P. forrestii*; c: *P. calophylla*; d: *P. graeca*; e: *P. laevigata*; f: *P. angustifolia*.
